# Development of Functional Gluten-Free Sourdough Bread with Pseudocereals and Enriched with *Moringa oleifera*

**DOI:** 10.3390/foods12213920

**Published:** 2023-10-26

**Authors:** Rocío Peñalver, Gaspar Ros, Gema Nieto

**Affiliations:** Department of Food Technology, Food Science and Nutrition, Faculty of Veterinary Sciences, Regional Campus of International Excellence “Campus Mare Nostrum”, University of Murcia, Espinardo, 30071 Murcia, Spain; rocio.penalver@um.es (R.P.); gros@um.es (G.R.)

**Keywords:** gluten-free bread, pseudocereals, *Moringa oleifera*, bioaccessibility, nutritional value

## Abstract

Celiac patients tend to have an unbalanced diet, because gluten-free products typically contain a high amount of fats and carbohydrates and a low amount of proteins, minerals, and dietary fiber. This research focused on the development of gluten-free functional breads using pseudocereals, psyllium, and gluten-free sourdough to replace commercial yeast, fortifying them with *Moringa oleifera*. Six different gluten-free breads were made with sourdough: three control breads differentiated by sourdough (quinoa, amaranth, and brown rice) and three breads enriched with moringa leaf differentiated by sourdough. The antioxidant capacity, phenolic compounds, nutritional composition, physicochemical parameters (color, pH, and acidity), folate content, amino acid profile, reducing sugars, mineral composition, mineral bioaccessibility, fatty acid profile, and sensory acceptability were evaluated. A commercial gluten-free (COM) bread was included in these analyses. Compared with COM bread, the reformulated breads were found to have better nutritional properties. Moringa leaf increased the nutritional properties of bread, and highlighted the QM (quinoa/moringa) bread as having increased protein, fiber, sucrose, glucose, maltose, phenylalanine, and cysteine. The AM (amaranth/moringa) bread was also shown to have a higher total folate content, antioxidant capacity, phenolic compounds, 9t,11t-C18:2 (CLA), and 9t-C18:1. Reformulated breads enriched with moringa could meet nutritional requirements and provide health benefits to people with celiac disease.

## 1. Introduction

Celiac disease is a chronic autoimmune enteropathy whose symptoms appear after the ingestion of gluten by genetically predisposed individuals, and is one of the most common lifelong disorders; the most effective treatment has proven to be strict adherence to a lifelong gluten-free diet. This diet, according to Vici et al. [1], is typically low in fiber, vitamin D, vitamin B12, folic acid, iron, zinc, and magnesium, with an unbalanced carbohydrate, fat, and protein intake [2]. People with celiac disease, as well as people with non-celiac gluten sensitivity and an increasing number of consumers who avoid gluten for lifestyle reasons, follow a gluten-free diet [3,4]. For this reason, the gluten-free food market has seen an increase in the development of gluten-free products in recent years [5].

Among gluten-free foods, bread is the most consumed; globally, it represents an important staple food product. Generally, gluten-free bread is a product with unsatisfactory sensory properties and a poor nutritional quality [6,7], as it is mainly based on starch and refined flours, resulting in products that are high in available starch and low in protein, dietary fiber, micronutrients, and bioactive compounds [8,9].

The pseudocereals buckwheat, quinoa, and amaranth have received much attention in recent years due to their nutritional profile and health benefits [10,11]. Pseudocereals contain a high protein content with a balanced amino acid composition and are also an important source of phytochemicals, dietary fiber, vitamins, and minerals [12]. In addition, buckwheat, quinoa, and amaranth are naturally gluten-free and are considered healthy ingredients for fortifying gluten-free foods [11,13].

Grain legume meals are a good source of phenolic compounds, as well as protein, carbohydrates, dietary fiber, minerals, and vitamins [14,15]. In a study conducted by Santos et al. [16], in which 75% of rice flour was replaced with chickpea flour, an improvement in bread quality and an increase in protein content, dietary fiber, resistant starch, reduced glycemic index, and satiety were reported.

*Moringa oleifera* is known as a miracle plant because of its high nutritional properties [17]. *Moringa oleifera* leaf has been used as an alternative food source to combat malnutrition [18], as it contains significant amounts of total phenols, calcium, iron, potassium, magnesium, manganese, functional peptides, folic acid, and copper [19]. In addition, incorporating *Moringa oleifera* leaf powder into bread is an effective strategy to enhance the daily intake of protein and fiber, all while utilizing a robust crop. Amidst the shifting dynamics of our global climate, the hardy *Moringa oleifera* tree demonstrates remarkable adaptability to emerging regions impacted by rising temperatures, such as the Mediterranean basin. These characteristics show promise for expanding the cultivation of this crop. Furthermore, previous research by Hussin et al. [20] recommended the use of *Moringa oleifera* leaf powder to augment the nutritional profile of food items, particularly with regards to minerals and amino acids.

One way to nourish the population would be through fortified foods. Thus, the addition of moringa to staple foods, such as bread, could be a safe way for both the healthy and celiac population to consume it [21]. Celiac patients have deficiencies in many micronutrients, including iron; folic acid; vitamins A, B6, B12, D, E, and K; calcium; and zinc, due to malabsorption [22,23]; in addition, gluten-free products contain lower levels of vitamins D, E, and B12; iron; folate; magnesium; potassium; and sodium than gluten-containing foods [1]. This lack of fortification could facilitate an increased risk of micronutrient deficiency in subjects with celiac disease on an adequate gluten-free diet [1]. According to Vici et al. [1], only 5% of gluten-free breads contain the four mandatory fortification micronutrients (calcium, iron, niacin, and thiamine), while 28% are fortified only with calcium and iron. However, it should be noted that not all countries require food fortification. In fact, some countries only require the fortification of wheat-based products, and these rules are not imposed on “dietetic or special foods”, such as gluten-free products. There have been some studies on the use of moringa leaf powder as a nutritional supplement to wheat bread, but there are no studies on gluten-free bread with added moringa leaf [17,24,25].

Gluten-free bread lacks the above-mentioned nutrients found in pseudocereals, pulses, and *Moringa oleifera* [26], which could thus be used to make and enrich gluten-free bread.

In addition, the nutritional enhancement resulting from sourdough fermentation has a significant influence on the availability of micronutrients, protein digestibility, phenolic compound concentration, antioxidant capacity, and functional characteristics (such as a low glycemic index) [27,28]. This has increased the interest of consumers who are focused on their well-being and health, thereby creating fresh market prospects for the bakery industry. Sourdough is a natural improvement that can be used in the production of gluten and gluten-free bread [29].

As mentioned above, within the food product ranges, gluten-free formulations have been reported to have a lower nutritional value compared with gluten-containing products [30]. Many resources have been invested into the development of gluten-free bread in an attempt to make gluten-free bread comparable to gluten-containing bread in terms of nutritional and sensory characteristics [3,31]. In addition to the challenge of the technicality of production, mainly due to the absence of viscoelastic gluten, which makes the entire baking process problematic [32,33]. To address the lack of gluten and simulate its viscoelastic behavior, the addition of hydrocolloids such as psyllium plays a strategic role in making gluten-free dough workable [34].

The gluten-free diet can be significantly improved using alternative cereals such as quinoa, amaranth, and buckwheat; substituting commercial yeast for sourdough; adding *psylium* to create the gluten-like structure; and fortifying the bread with *Moringa oleifera*.

The aim of this research is to develop and manufacture a novel gluten-free functional bread that promotes the health of celiac patients. This bread is created by incorporating *Moringa oleifera* into amaranth, buckwheat, and chickpea flour, with the addition of three different sourdoughs: amaranth, buckwheat, and rice. The formulation of this bread is designed to meet the criteria needed for labeling it as both “a source of protein” and “high in fiber”. The study involves evaluating the nutritional composition, bioavailability, and sensory appeal of the enriched bread, as well as calculating the bioavailability of minerals.

## 2. Materials and Methods

### 2.1. Materials

Sugar, olive oil, and salt were purchased at local markets in Murcia, Spain. Amaranth flour was obtained from EcoAndesImportExport, Madrid, Spain. Psyllium, chickpea flour, and maize starch were procured from Planeta Huerto, Alicante, Spain. Buckwheat flour and brown rice flour were supplied from Biovitagral, Italy. Quinoa flour was obtained from Legumbres Pedro, Cádiz, Spain. Water was taken from the tap. Moringa powder obtained from 100% dried moringa leaves was supplied by KeyPharm Laboratories (Oostkamp, Belgium). *Moringa oleifera* contains significant amounts of total phenols (32.90 mg GAE/g), calcium (1.48 g/100 g), iron (25.14 mg/100 g) DW, potassium (1.75 g/100 g DW), magnesium (301.11 mg/100 g DW), manganese (7.21 mg/100 g DW), folic acid (16.57 g/100 g DW), copper (0.45 mg/100 g DW), protein (25.30 g/100 g DW), and fiber (24.97 g/100 g DW) [35]. Commercial gluten-free bread was purchased at the local supermarket (Hipercor, S.A., El corte ingles, Murcia, Spain) for comparison with the developed breads. Commercial bread is composed of the following ingredients: corn starch, water, sourdough (rice flour, water) 16%, rice starch, vegetable fiber (psyllium), rice syrup, sunflower oil, soy protein, thickener of hydroxypropyl methylcellulose, 1.9% millet flour, 1.3% quinoa flour, yeast, honey, and iodized salt (salt and potassium iodide).

### 2.2. Methods

#### 2.2.1. Sourdough Preparation

Three types of sourdoughs were prepared, differing in the type of flour: quinoa flour, amaranth flour, and brown rice flour. A spontaneous ferment was performed, which consisted of adding a 50:50 ratio of water and flour using a backsloping procedure every 24 h for 5 days at room temperature (25–26 °C), according to the method followed by Tomić et al. [36].

#### 2.2.2. Gluten-Free Bread Preparation

Gluten-free bread formulations are given in Table 1. Six different breads were prepared, three control breads differing in the sourdough—each bread created with quinoa sourdough, amaranth, or brown rice. The other three breads were prepared in the same way, but moringa was added at a concentration of 6% in relation to the flour. Bread ingredients were mixed to a homogeneous and fluid dough consistency in a mixer (Taurus, Giro, Oliana, Spain) for 5 min, followed by dough fermentation at 26 °C with 85% relative humidity for 5 h. The fermented dough was baked at 175 °C for 25 min (Fagor innovation 6H-757CX, Murcia, Spain). After baking, the loaves were allowed to cool for 1 h and were then vacuum packed in polyethylene bags.

#### 2.2.3. Chemical Composition

The proximate composition of the breads was determined using the standard methods of Association of Official Analytical Chemists (AOAC): moisture (964.22:1995), protein (955.04:1995), total fat (920.390:1995), ash (923.03:1995), total dietary fiber, soluble fiber, and insoluble fiber (985.29:1985). Carbohydrates were calculated by difference according to the following formula:

Carbohydrates = 100 − (total fat + ash + protein + moisture + total dietary fiber).

#### 2.2.4. pH, Total Titratable Acidity (TTA), and Acid Content

pH was measured in triplicate and was determined with the help of a pH meter (Crison GLP21) after mixing 10 g of fresh bread sample with 90 mL of distilled water at room temperature. The total titratable acidity (TTA) was then measured by titration with 0.01 NaOH and was expressed as ml NaOH/10g.

Concentrations of L-lactic and acetic acid were analyzed using a specific kit Acetic Acid Assay Kit (Acetate Kinase) and L-Lactic Acid Assay Kit (L-Lactate dehydrogenase) (Byosistems S.A, Barcelona, Spain), respectively, according to manufacturer’s instructions. The results were expressed as g of L-lactic or acetic acid per kilogram of bread. Three replicates were averaged for each sample.

#### 2.2.5. Color

Color was measured using a Konica Minolta CR-410 chromameter (Minolta Camera Co., Osaka, Japan) and the DP-400 data processor of “AQ instruments” was used to measure crust color (CIE Lab* values). CIE L* values (lightness), CIE C* values (saturation), a* values (red-green), b* values (yellow-blue), and h* (nuance) were measured. Three replicates were averaged for each sample.

#### 2.2.6. Antioxidant Capacity

The bioactive compounds of the breads were extracted in 80% aqueous methanol, as reported by Repo-Carrasco and Encina-Zelada [37], with some modifications. The bread (2 g) was first homogenized in 8 mL of 80% methanol and then left under refrigeration 4 °C for 24 h in the dark. After 24 h, they were centrifuged at 4500 rpm, 4 °C for 25 min. Finally, they were filtered with 0.45 mm nylon filters and stored at −20 °C until analysis. The extraction of bioactive compounds from the breads was performed in triplicate.

Four assays were used to determine the antioxidant activity of the breads based on antiradical activities: (1) radical scavenging activity capacity (DPPH), (2) cation radical scavenging activity (ABTS), (3) reducing ferric antioxidant power (FRAP), and (4) oxygen radical scavenging capacity (ORAC).

Antioxidant activity related to the chelating capacity was measured using the 2,2-diphenyl-1-picrylhydrazyl (DPPH) free-radical scavenging method described by Brand-Williams et al. [38] and Sánchez-Moreno et al. [39]. The extract (100 µL) was mixed with 3.9 mL of DPPH reagent and allowed to stand for 30 min before reading at 515 nm absorbance.

The radical cation scavenging activity (ABTS) was determined by mixing 100 µL of extract with 1 mL of 7 mM 2,2-azinobis(3-ethylbenzothiazolin)-6-sulfonic acid (ABTS) reagent with an absorbance of 0.70, prepared according to Re et al. [40]. The absorbance of the mixture was measured at 734 nm after reacting for 2 min at room temperature.

The ferric reducing antioxidant power (FRAP) assay was performed by mixing 100 uL of extract with 1 mL of FRAP reagent for 4 min at 37 °C under dark conditions, as reported by Benzie and Strain [41]. The FRAP reagent was performed daily with a solution of 800 mM acetate buffer, pH = 3.6, 20 mM FeCl_3_–6 H_2_O, 10 mM TPTZ (2,4,6-tripyridyl-s-triazine) in 40 mM (10:1:1, *v*/*v*/*v*). The absorbance of the mixture was measured at 593 nm against a blank.

The oxygen radical absorbance capacity (ORAC) method [42] was followed to measure the hydrophilic antioxidant capacity. The reaction was performed in a phosphate buffer (0.075 M, pH 7.0). For this purpose, 20 μL of the extract, at different concentrations, and 20 μL Trolox standards, were pipetted into a black 96-well plate. After plate preparation, 200 μL of fluorescein was dispensed into each well. After 15 min at 37 °C in dark incubation, the reaction was initiated by adding 20 μL of 40 mM AAPH (2,2′-azobis(2-amidinopropane) dihydrochloride) to each well using the Synergy HT plate reader after a purge with water and the corresponding reagents. Fluorescence decay was measured using the Biotek Synergy HT plate reader at 485 nm excitation and 528 nm emission every minute for one and a half hours at 37 °C. The antioxidant activity of the sample was expressed as µM of Trolox equivalents (TEs) per g of extract.

#### 2.2.7. Total Phenolic Content (TPC)

The determination of the total phenolic compounds was carried out using the Folin−Ciocalteu method described by Singleton and Rossi [43] using Folin−Ciocalteu reagent and gallic acid as the standard (20, 40, 60, 80, and 100 mg/L). The absorbance of the extracts was measured at 750 nm. The analysis was performed in triplicate and the TPC were expressed as mg gallic acid equivalents (GAEs) per g of sample.

#### 2.2.8. Determination of Disaccharides and Monosaccharides

The sucrose, maltose, D-glucose, and D-fructose concentrations were analyzed using three specific kits: maltose/sucrose/D-glucose/D-fructose, sucrose/D-glucose/D-fructose and D-glucose/D-fructose (Byosistems S.A, Barcelona, Spain), respectively, according to manufacturer’s instructions. The analysis was carried out on the Biosystems Y15/c (Byosistems S.A, Barcelona, Spain) multiparameter automatic analyzer. The results were expressed as g of maltose, sucrose, D-glucose, or D-fructose per 100 g of bread. Three replicates were averaged for each sample.

#### 2.2.9. Bioaccessibility and Determination of Minerals

For the evaluation of the mineral bioaccessibility, simulated gastrointestinal digestion of the samples was performed using the method proposed by Minekus et al. [44]. For this purpose, three simulated digestion fluids were performed: salivary (SSF), gastric (SGF), and intestinal fluid, for the three phases of digestion (oral, gastric, and intestinal). Starting with the oral phase, the bread was mixed with SSF electrolyte stock solution (50:50; *w/v*) and the pH was adjusted to 7. Human salivary amylase (75 U/mL) and 0.75 mM CaCl_2_ were added. Once ready, it was incubated at 37° for 2 min. For the gastric phase, the above mixture was mixed with SGF to obtain a final ratio of 50:50 (*v/v*) and the pH was modified to 3.0 using 6 M HCl. Porcine pepsin (2000 U/mL) and 0.075 mM CaCl_2_ were added, after which the samples were incubated at 37 °C for 2 h. Finally, for the intestinal phase, SIF was added to the obtained mixture (50:50) and 0.3 mM CaCl_2_. The pH was neutralized with 1 M NaOH and porcine pancreatin and bile salts were added at 100 U/mL and 10 U/mL, respectively. The aliquot was divided into two: one was called the soluble phase and the other the dialysate phase, which was introduced through a dialysis membrane. Both phases were incubated at 37 °C for 2 h. The supernatant obtained was filtered through 0.2 m and the mineral content was measured by inductively coupled plasma mass spectrometry (ICP-MS) (Thermo electron X7 inductively coupled plasma mass spectrometry, model X series, Agilent, Santa Clara, United States). The standards were diluted and used to calibrate the ICP-MS for mineral analysis in the samples studied [45].

#### 2.2.10. Amino Acid Content

Amino acids were extracted following the method proposed by Shahidi and Synowiecki [46]. Amino acids were derivatized using 6-aminoquinolyl-N-hydroxysuccinimidyl carbamate (Waters AccQ-Fluor reagent kit) and determined using HPLC (Waters 2695 Separations Module + Waters 2475 Multi Fluorescence Detector + Waters AccQ-Tag amino acids analysis column). Tryptophan was destroyed during amino acid analysis and was thus not quantified. The amino acid content was expressed as mg/100 g dry matter.

#### 2.2.11. Fatty Acid Profile

The lipids extracted (50 mg) were used to determine the fatty acid profile. The total fatty acids were transesterified using the method previously described by Domínguez et al. [47]. A GC equipment (GC-Agilent 6890 N; Agilent Technologies Spain, S.L., Madrid, Spain) with a flame ionization detector was used for the separation and quantification of the fatty acid methyl esters (FAMEs) using the chromatographic conditions proposed by Domínguez et al. [47]. Individual FAMEs were identified by comparing their retention times with those of authentic standards (Supelco 37 component FAME Mix, Sigma-Aldrich, Barcelona, Spain). C18:1n-7 cis (Supelco cis-11-Vaccenic methyl ester), C18:1n-11 trans (trans-11-vaccenic methyl ester), and C18:2n-7 (CLA) (Matreya LLC Methyl 9(z),11 (E)-octadecadienoate) were not included in the commercial mix. In addition, nonadecanoic acid (C19:0) was used as the internal standard, which was added to the samples prior to methylation. Data were expressed in g/100 g of FAME.

#### 2.2.12. Determination of Folates

Folates were extracted from the sample following the procedure described by Konings and Pfeiffer et al. [48,49]. For that, 1 g of sample was mixed with 25 mL of extraction buffer (50 mmol/L CHES, 50 mmol/L HEPES, containing 2 g sodium ascorbate/100 mL and 10 mmol/L 2-mercaptoethanol, pH 7.85) under a nitrogen atmosphere. The extraction mixtures, in screw-capped tubes, were placed in a boiling water bath for 10 min, cooled on ice, and homogenized. Then, the pH was adjusted to 4.9 with 60 mmol/L HCl. Enzymatic des conjugation and purification of samples were carried out following the methodology described by Vahteristo et al. [50]. An aliquot of 5 mL was incubated for 3 h at 37 °C under a nitrogen atmosphere with 1 mL of α-amylase preparation (20 mg/mL in 1 g sodium ascorbate/100 mL) and 1 mL of hog kidney conjugase prepared from fresh pig kidneys, as described by Gregory, Sartain, and Day [51]. To inactivate the enzymes, the samples were boiled at 100 °C for 5 min. The samples were centrifuged at 2000 rpm for 10 min and filtered on 0.45 μm pore size filters and passed through purified, strong anion exchange (SAX) cartridges connected to a Supelco 12-port vacuum manifold.

The separation and analysis of samples were performed with an HPLC/MS system consisting of an Agilent 1290 Infinity II Series HPLC, as described by López-Nicolás et al. [52]. The folate standards used were trihydrochloride tetrahydrofolic acid (H4), 5-Methyltetrahydrofolic acid, 5-formyltetrahydrofolic acid, and folic acid supplied by Dr. Schirck’s Laboratory (Jona, Switzerland).

#### 2.2.13. Sensory Analysis

The sensory analysis was carried out by means of hedonistic tasting involving 35 untrained panelists aged 19–59 years. The analysis comprised the evaluation of the seven different types of breads, which were coded with three random digits. It was conducted in isolated booths according to ISO-8586 [53]. The booths were separated from each other in order to isolate the study participants, thus avoiding the possible influence of the perception of the other panelists. The parameters of appearance, color, aroma, texture, juiciness, taste, purchase intention, and general acceptance were evaluated on a scale of 1 to 5, with 1 being the worst (‘I don’t like it at all’) and 5 being the best (‘I like it very much’).

#### 2.2.14. Statistical Analysis

All statistical analyses were performed using IBM SPSS Statistics^®^ 28 software (IBM Corporation, Armonk, NY, USA). The proximate composition, amino acids, fatty acids, mineral bioaccessibility, folates, antioxidant capacity, and phenolic compounds among the breads studied were examined using two-way ANOVA tests for the difference between the type of sourdoughs and for the difference between the addition of moringa. Least squares means were compared using the post hoc HSD Tukey test (significance level *p* < 0.05). Values were given in terms of mean values ± standard deviations. Person’s correlation coefficients was used to evaluate the relationship between physicochemical parameters (pH and acidity) and acid contents (lactic and acetic) of bread.

## 3. Results and Discussion

### 3.1. Chemical Composition

The chemical compositions of the gluten-free breads samples are presented in Table 2. As for moisture, COM bread (42 g/100 g) was found to have the highest moisture content and Q bread (32.34 g/100 g) the lowest.

It was observed that with the inclusion of moringa leaf, the ash content increased significantly compared with the control breads, especially in the QM bread (2.33 g/100 g). The COM bread (0.01 g/100 g) obtained the lower ash content compared to formulated breads. Aly et al. [54] also found that the inclusion of moringa leaf in bread increased the ash content. This finding also coincides with that obtained by Sardabi et al. [55], where they added moringa seed to wheat bread. Regarding the control breads, a higher ash content was observed than the in the commercial bread; this is mainly because buckwheat, amaranth, and quinoa are pseudocereal that stand out for their mineral content, and it is already known that the ash content is related to the level of minerals [56].

Regarding total fat, the COM bread (3.25 g/100 g) was found to have the highest content compared with the formulated breads. The inclusion of the moringa leaf showed a significant increase in fat content compared with the control breads. Aly et al. [54] also found that adding moringa to bread increased the fat level of the bread; this is because moringa leaf contains 5–6 g/100 g Dw of total fat [35]. A previous study indicated that moringa leaf contains a high proportion of polyunsaturated fatty acids [57]; therefore, moringa leaf is recommended for human consumption as the polyunsaturated fatty acids contents include omega-3 and -6, which are fatty acids that have been shown to help prevent and treat cardiovascular and neural diseases [58,59,60]. In the formulated breads, breads with quinoa sourdough stood out in both the control and enriched breads; this is because the fat content of quinoa (6.07 g/100 g) is higher than that of brown rice and amaranth [28].

Regarding protein, in the control breads no significant differences were found between the different sourdoughs, highlighting the highest level in the quinoa sourdough and the lowest level in the brown rice sourdough. However, the enriched breads did show significant differences between the different sourdoughs, with the bread with quinoa sourdough (QM) standing out with the highest protein content (6.76 g/100 g) as in the control breads. Therefore, the quinoa sourdough contributed protein to the bread, due to the high protein content of quinoa [61].In addition, the sourdough increased protein digestibility due to proteolysis produced during the fermentation period [62]. In the enriched bread, the protein level was much higher, as *Moringa oleifera* is high in protein (25.30 g/100 g Dw) [35]. The gluten-free bread with the lowest protein content significantly was COM bread (2.96 g/100 g), due to the high proportion of refined flours with a low level of protein [63].

The highest content of total, insoluble, and soluble dietary fiber was obtained in the QM bread and the lowest was in the COM bread. In both the control and enriched breads, a higher content of total, soluble and insoluble dietary fiber was observed in the breads with quinoa sourdough, because quinoa is the pseudocereal with a highest content of total, insoluble, and insoluble fiber compared with the other pseudocereals and cereals present in this study. This is mainly caused by lactic acid bacteria, which contain proteinases that result in the progressive hydrolysis of the cereal or pseudocereal [64]. Both the control and enriched bread with brown rice sourdough showed the lowest total and soluble fiber content. According to Yan et al. [65], brown rice contains 1.27–1.69 g/100 g of soluble fiber and 4–5 g/100 g of total fiber, which is much lower compared with the soluble (1.8–3.7 g/100 g) and total (7.3–11.4 g/100 g) fiber in amaranth and the soluble (2.1–3.9 g/100 g) and total (7–9.5 g/100 g) fiber in quinoa [64]; however, concerning insoluble fiber, the lowest content was found in bread with amaranth sourdough (A) (6.80 g/100 g), slightly lower than that found in bread with brown rice sourdough (BR). A significant increase in total, insoluble, and soluble fiber was observed in the breads enriched with moringa, mainly due to the high fiber content of the moringa leaf powder (24.97 g/100 g) [35]. This finding was also observed by Nudel et al. [66] when adding moringa leaf in different concentrations to wheat bread. The bread with the lowest fiber content was the COM bread, mainly due to the high proportion of refined flours—flours that are characterized as starchy foods that are low in dietary fiber [6].

Carbohydrates depend on the amount of moisture, ash, fat, fiber, and protein. The highest carbohydrate content was obtained in the A bread (48.98 g/100 g), and the bread with the lowest level was the AM bread (36.27 g/100 g). Breads enriched with moringa had a higher content of lipids, ash, fiber, and protein, which resulted in a decrease in carbohydrates in these breads. This was also observed by Sardabi et al. [55], who found that the addition of moringa seed to wheat bread reduced its carbohydrate content. Ajibola et al. [67] also agreed with the finding, where they prepared different cookies with moringa leaf in percentages of 0–10% and obtained a reduction in carbohydrates with an increase of 3–5% moringa to the cookie. But, in addition to the incorporation of moringa leaf, this decrease in carbohydrates may also be due to the sourdough, as several studies have observed a decrease of at least 30% fermentable polyols in sourdough breads, which will result in a lower amount of fermentable carbohydrates and free glucose [68,69].

### 3.2. pH, Total Titratable Acidity (TTA), and Acid Content

The pH, acidity, L-lactic acid, and acetic acid contents are shown in Table 3. Regarding pH, it was observed that the COM bread obtained the lowest pH and the highest pH was obtained by the BR bread; however, it was found that the breads enriched with moringa leaf had significantly lower pH values (*p* < 0.05) than the control breads. Among the different types of sourdough used, a slight variation could be observed, with the lowest pH levels found in the Q and A breads and the highest in the BR bread. This is mainly as a result of the pH levels of amaranth and quinoa, which are approximately 5, while the pH of brown rice is close to 3 [70]. The total titratable acidity values were affected by the presence of moringa leaf, causing an increase in the ml of NaOH spent, therefore increasing the acidity of the gluten-free bread. These results are in agreement with the pH presented by each sample. This data coincide with the study of Faten Dhawi on buffalo yogurt, where a slight increase in yogurt TTA was observed when *Moringa oleifera* was added to its composition [71].

According to the legislation on bread, RD 308/2019 [72] states that a bread with sourdough must have a pH value of less than 4.83. Therefore, the newly formulated breads would not be within the legislation. However, it is perhaps not possible to use this as a comparison as we used gluten-free sourdough bread, and there is currently no specific legislation for gluten-free bread.

The addition of moringa leaf significantly affected (*p* < 0.05) the lactic and acetic acid contents in the breads. This effect may be due to the high mineral content of *Moringa oleifera* [73], as it has been observed that a high mineral content increases the buffering capacity of the sourdough system, which increases lactic acid generation [74]. The breads containing sourdough showed higher lactic and acetic acid than the commercial bread. Lactic and acetic acids are the main metabolites of sourdough processes; lactic acid bacteria are part of the stable microbiota of mature sourdoughs [75].

Strong correlations for the following were found between the variables of total titratable acidity (TTA), acetic acid and lactic acid. A positive correlation was observed between lactic acid content and TTA (r = 0.552, *p* = 0.010) and between acetic acid content and TTA (r = 0.793, *p* < 0.001), indicating that an increase in the content of lactic acid and acetic acid increases the acidity of the bread.

### 3.3. Color

The colorimetric parameters (lightness coordinate (L*), redness coordinate (a*), yellowness coordinate (b*), saturation (C*), and tone (h*)) measured in the breads are reported in Table 4. The highest brightness value was observed in the COM bread (84.39 ± 1.49), due to its high starch and rice flour content [76]. A significantly lower value was observed for parameter a* in the COM bread.

**Figure 1:**
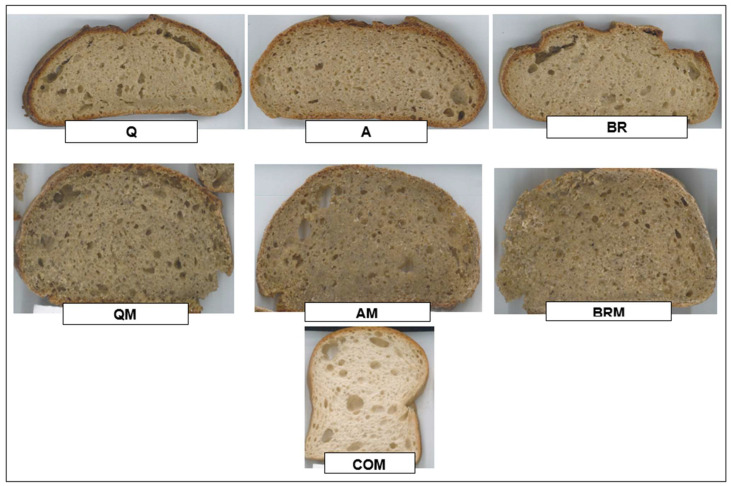
Photograph of the enriched gluten-free, control, and commercial breads. Q: sourdough quinoa; A: sourdough amaranth; BR: sourdough brown rice; QM: sourdough quinoa and *Moringa oleifera*; AM: sourdough amaranth and *Moringa oleifera*; BRM: sourdough amaranth and *Moringa oleifera*; COM: commercial.

The addition of moringa leaf affected the color parameters of the bread samples (Figure 1). With the inclusion of moringa leaf in the bread, a significant decrease (*p* < 0.05) was observed in the L* and h parameters; however, on the contrary, a significant increase was observed in the a* value of the breads enriched with moringa. In the C and b* values, no significant differences were found between samples, but it was observed that a slight decrease was obtained with the addition of moringa leaf. Similar results were obtained by Sardabi et al. [55], where the addition of moringa seed observed modifications in the L*, b*, and a* parameters of the bread compared with the controls. These results were also consistent with a study by Govender and Siwela [77], who found that as the amount of moringa leaf increased, the brightness decreased and the value of a* increased.

Generally, color changes in the bread formulated with moringa leaf depend on the presence of pigments in *Moringa oleifera*, particularly because of the high chlorophyll content (1.46 mg/g) [78]. In fact, the darkening observed when adding moringa leaf and using pseudocereals can be considered a positive point, as gluten-free breads are usually characterized by a poor color compared with breads that have gluten.

### 3.4. Antioxidant Activity

The total phenolic content (TPC) and antioxidant activity of the seven breads is summarized in Table 5. The TPC and antioxidant activity of the breads were significantly increased with the addition of moringa leaf. In fact, regarding TPCs, with the inclusion of moringa leaf, an increase of 64.54% was observed in the quinoa sourdough bread, an increase of 96.37% was observed in the amaranth sourdough bread, and an increase of 71.15% was observed in the brown rice sourdough bread. This same finding also coincides with Páramo-Calderón et al. [79], who observed that the inclusion of 1–3% moringa flour increased the amount of phenolic compounds in dry tortillas.

All six of the formulated gluten-free breads showed significantly (*p* < 0.05) higher TPC compared with the other commercially available bread. The control breads with sourdough were found to have a higher content of phenolic compounds than the commercial bread, mainly because the commercial bread was fermented with 16% sourdough and commercial yeast. According to Garzon et al. [80], sourdough improves the extraction of free phenols. In addition, because of the high content of phenolic compounds in pseudocereals, such as buckwheat, quinoa, and amaranth [80], the control breads and those with amaranth and quinoa sourdough moringa obtained higher levels of phenolic compounds. The moringa-enriched breads were much higher in TPC content, due to the high TPC content in the dried moringa leaf and the wide range of phenolic compounds it contains [35,81].

The antioxidant activity of the moringa-leaf-enriched, control, and commercial gluten-free bread was determined using ORAC, DPPH, ABTS, and FRAP methods. For all of the methods, in general it was observed that the antioxidant capacity was significantly low in commercial bread (1.34–436.25 µmol TE g-1) compared to formulated breads. With respect to the formulated bread, in all methods, a significant increase was observed with the inclusion of moringa leaves, standing out with greater antioxidant capacity in the AM bread (9.37–1235.52 µmol TE g-1). This high antioxidant capacity was due to the high antioxidant capacity of amaranth; in fact, it is one of the five pseudocereals with the greatest antioxidant potential [82,83]. This excellent antioxidant capacity of amaranth is due to the presence of phenolic acids, flavonoids, phytosterols, and squalene [84]. In the enriched breads, the antioxidant capacity increased by 27.56–53.6% in the quinoa sourdough bread, the amaranth sourdough bread showed an increase of 24.18–61.15% and the brown rice sourdough bread showed an increase of 7.13–64.97%. These increases in antioxidant activity were related to the increase in TPC. *Moringa oleifera* is a plant that, in addition to containing polyphenols such as phenolic compounds, β-carotene, ascorbic acid, chlorogenic acid, gallic acid, kaempferol, and quercetin glycosides [85,86,87], contains other compounds with antioxidant activities, such as carotenoids, vitamins C and E, and several micronutrients such as selenium and zinc [79].

From these data, it can be determined that the breads enriched with moringa, especially the AM bread, had the highest TPC content and antioxidant capacity, probably due to the high content of total moringa compounds, 32.90 mg GAE/g DW [35], and the variety of bioactive compounds contained in amaranth, such as p-hydroxybenzoic acid, rutin, vanillic, and gallic acids [61].

### 3.5. Sugars

Table 6 shows the amount of reducing sugars (fructose, glucose, and maltose) and sucrose in the breads. For glucose (0.78 g/100 g) and maltose (2.74 g/100 g), the highest content was observed in commercial bread (*p* < 0.05). However, for fructose, the highest content was found QM bread (0.15 g/100 g) and AM bread (0.15 g/100 g), and in sucrose it was found in the QM bread (2.56 g/100 g).

Among the breads with sourdough, higher contents of sucrose and fructose were found in the breads made with quinoa sourdough, followed by amaranth sourdough. This was mainly due to the high content of these sugars in the quinoa and amaranth pseudocereals [81,88]. In contrast, more glucose was observed in the breads with less sourdough compared with the COM bread, as a preliminary study showed that bread with sourdough reduced glucose as a result of natural fermentation and baking [89]; so, it could be used to prevent the onset of type 1 diabetes [90].

In general, all sugars (glucose, maltose, fructose, and sucrose) showed a slight increase or similar values to the control in the breads with the addition of moringa. This increase was due to the presence of glucose, fructose, and sucrose in moringa leaves [91,92,93].

### 3.6. Minerals Content and Bioaccessabilty

The results of the minerals are shown in Table 7. No As, Be, Cd, Co, La, Li, Mo, Se, Tl, V, and Ti were found in the samples and no differences were seen between the samples regarding the amount of Al, Bi, and Cr.

In general, an increase in all minerals was observed with the addition of moringa leaves. This increase in minerals through the inclusion of moringa was also found by Govender and Siwela [77], where moringa was added to white and brown bread. In fact, this increase was even observed with the addition of moringa leaves in commercial baby foods [94].

High boron, calcium, and manganese contents were observed in the AM bread, with the lowest found in the Q bread (*p* < 0.05). This was mainly due to the high content of these minerals in moringa leaves and amaranth [95,96]. Boron was not detected in the COM bread. The highest content of these zinc and lead was found in the QM bread (7.24 mg/Kg; 0.11 mg/Kg) and the lowest content was found in the COM bread (0.01 mg/Kg; 3.70 mg/Kg; 0.05 mg/Kg). A higher amount of zinc was found in the formulated breads than in the commercial bread (COM); this may be due to the fact that moringa leaves and pseudocereals containing a significant high amount of zinc [28,91,97]. The QM bread had the highest content of the minerals potassium, sulfur, and sodium, because quinoa is a pseudocereal rich in potassium and moringa is rich in sodium [28,97,98]. However, the lowest iron and sulfur contents were observed in the BR bread, where, in addition to the low content in the BR bread, the same lower content was found in the A and Q breads. The lowest potassium content was found in the COM bread (0.31 g/100 g) and the lowest sodium content was found in the Q bread (0.27 g/100 g), compared with AM bread, which had the highest iron (7.54 mg/Kg) and magnesium content (0.04 g/100 g). This may be because is pseudocereal rich in iron and magnesium (6.36 mg/100 g; 237.39 mg/100 g) [28], and also because moringa leaves are also rich in iron and magnesium [91].

The minerals of greater interest in celiac patients are calcium and iron, as these are common deficiencies in this population [99]. In calcium, an increase of 26.67–41.38% was observed in the breads enriched with moringa leaves compared with the control breads, because moringa leaves contain a high calcium content (1.48 g/100 g) [35]. In iron, an increase of 3.83–96.87% was observed in moringa-enriched breads compared with the COM bread; and of 20.89–54.91% compared with the control breads compared to COM bread. This is mainly because iron is naturally found in moringa leaves (25.14 mg/100 g) [35], quinoa (3.28 mg/100 g), brown rice (0.46 mg/100 g), amaranth (6.36 mg/100 g), and buckwheat (2.26 mg/100 g) [28]. This finding was also found by Govender and Siwela [77], where they observed an increase in calcium and iron when moringa leaves were added to white bread, but the increase observed was much higher, with calcium increasing by 150–275% and iron by 16.92–154.62%.

The World Health Organization (WHO) recommends consuming 250 g of bread per person per day [100]. According to the AESAN, the nutritional intakes of calcium in adults in women and men range between 1000–1300 mg/day [101], taking into account that 250 g of bread should be consumed daily, with breads enriched with moringa leaf, 950–1150 mg/day would be obtained, which would provide a large part of the daily nutritional intake. Regarding iron, the daily nutritional intake in adults ranges between 9.1–18 mg/day [101], and as 250g/day of bread with bread enriched with moringa leaves would provide between 1.17–1.89 mg, it would not provide a significant amount of iron, but it would help with the daily nutritional intake of iron.

**Figure 2:**
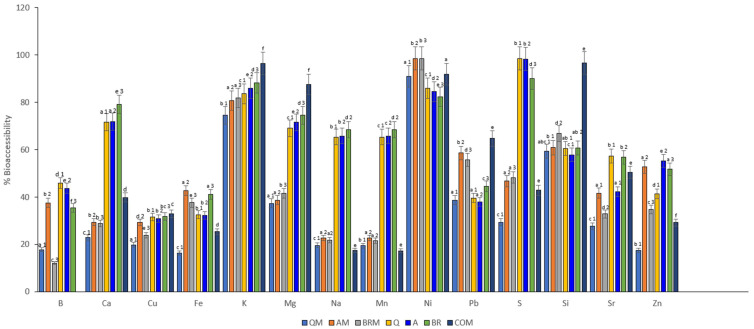
Percentage of bioaccessible minerals in the studied formulas of the gluten-free breads. Different letters denote significant differences among gluten-free breads (*p* < 0.05). Q: sourdough quinoa; A: sourdough amaranth; BR: sourdough brown rice; QM: sourdough quinoa and *Moringa oleifera*; AM: sourdough amaranth and *Moringa oleifera*; BRM: sourdough amaranth and *Moringa oleifera*; COM: commercial. Different subscript letters indicate significant differences (*p* < 0.005) between samples ^a–f^. Different subscripts indicate differences between samples depending on the sourdough ^1–3^.

Based on these data and the data obtained, the bioaccessible fraction (in percentage) of each mineral was calculated and the results are shown in Figure 2. As can be seen, the most bioaccessible mineral was Ni, followed by K, Si, S, Mg, Ca, Pb, Sr, Na, Mn, Fe, Cu, B, and Zn. Significant differences were observed in the enriched breads and control breads compared with the COM bread for most minerals, except for Cu and Ni. In fact, the COM bread presented ~100% higher bioaccessibility for K and Si, ~90% for Mg, and ~70% in Pb compared with the rest of the reformulated breads. On the contrary, the enriched samples presented higher bioaccessibility (~100%) for Ni. This is due to the fact that moringa leaf contains high levels of phytic acid (0.00182 mg/100 g) and tannin (0.22 mg/100 g); both compounds have a strong chelating capacity, and are generally combined with calcium, zinc, magnesium, potassium, and other minerals to give rise to insoluble salts associated with protein molecules and leading to their precipitation [91], producing a reduction in mineral bioavailability. On the one hand, it was observed that the control breads with sourdough were more bioaccessible in minerals Ca, Fe, B, Na, Mn, S, Sr, and Zn, compared with the enriched and commercial breads; this is due to the fact that besides influencing the absorption of minerals, phytates and tannins also limit the absorption of minerals and dietary fiber [102], so it is consistent that the enriched with moringa leaf were less bioaccessible, as more dietary fiber was obtained in the enriched breads. In addition, higher bioavailability was also obtained mainly because sourdough decreases the level of phytate in the bread, due to the low pH level during fermentation, which stimulates the phytase of the pseudocereal grain and the phytase activity of lactic acid bacteria [103,104].

But, on the other hand, it was observed that among the control breads with sourdough, the bread with the least bioaccessibility was quinoa sourdough, followed by amaranth and, finally, brown rice; this is mainly due to the amount of phytates present in quinoa [97].

From these data, it can be determined that the control breads with sourdough, especially BR bread, obtained higher bioaccessibility of Fe and Ca minerals, which are important minerals in celiac patients due to their malabsorption [105]. The BR bread obtained greater bioaccessibility of these minerals mainly due to the low content of phytic acid, and it is also corroborated in Pedro’s study that brown rice has a bioavailability of 35.31–61.68% for iron [106].

### 3.7. Amino Acid Profile

Table 8 shows the amino acid (AA) composition of gluten-free breads. The most abundant amino acid in gluten-free breads was Glu, followed by Asp, Arg, Pro, Phe, Leu, Lys, Gly, Ser, Val, Thr, Ala, Hys, Ile, Tyr, Cys, Met, and Hyp. The control breads showed higher total, total essential, and non-essential amino acid contents (all amino acids are represented in Table 8), except for methionine, phenylalanine, cysteine, and hydroxyproline, which were found to have a higher content in the breads enriched with moringa leaf. The highest phenylalanine and hydroxyproline content was found in QM bread (543.54; 5.08 mg/100 g). This is mainly due to the fact that quinoa has a high phenylalanine content and moringa leaves also have a high phenylalanine content [107,108,109]. However, higher levels of methionine were found in the AM bread, due to the high methionine content in amaranth [110]. Regarding cysteine, a higher content was observed in BRM bread, which may be due to the fact that rice contains a significant amount of cysteine (1.43–7.43 mg/g) [111].

Commercial bread had lower values for all amino acids compared with the control breads, except for methionine (66.79 mg/100 g), phenylalanine (213.73 mg/100 g), cysteine (69.04 mg/100 g), and hydroxyproline (0.26 mg/100 g). These high values in the formulated breads were mainly due to the high content of amino acids in quinoa, buckwheat, and amaranth [112].

In general, it was observed that enriched breads decreased in their amino acid content with the addition of moringa leaf. In this sense, Hussin et al. [20] also found the same coincidence, where, when moringa was added to noodles, the content of most amino acids decreased. Moringa leaf is an important source of phenolic compounds [90] that, according to previous findings, could form clusters (crosslinks) with these amino acids to protect them against thiol loss and thus protein oxidation, which has already been demonstrated with tea catechins [113] and phenols from rosemary and oregano [114,115]. This theory could justify the reduction in amino acid content in breads enriched with moringa leaf. Even so, the enriched breads and control breads are a good alternative to include in the gluten-free diet for the intake of essential amino acids, as these must be incorporated into the body through dietary proteins as they are not synthesized by mammals [116].

### 3.8. Fatty Acids Profile

The fatty acid profiles are shown in Table 9. Overall, monounsaturated fatty acids accounted for 50% of the total fat content, while polyunsaturated fatty acids accounted for 30% and saturated fatty acids for 20%. Of the monounsaturated fatty acids in the samples, the most abundant were as follow: palmitoleic acid was found in the range of 3.57–30.49 mg/100 g (COM and BR), elaidic acid in the range of 0–1.68 mg/100 g (A, BR, Q, and AM), oleic acid (C18:1n-9) in the range of 909.10–2045.27mg/100 g (COM and BR), cis-11-eicosenoic acid in a range of 6.18–18.10 mg/100 g (COM and Q) and erucic acid in a range of 1.55–6.90 mg/100 g (BR and QM).

Regarding polyunsaturated fatty acids (PUFAS) linoleic acid was the greatest at 94%, followed by α-linolenic acid at 4.5%, cis-9, trans-11-linoleic acid at 0.4%, and cis-11,14-eicosadienoic acid at 0.16%.

In this regard, in Table 9, the main fatty acids found in the gluten-free breads were linoleic (C18:2n-6), palmitic (C16:0), stearic (C18:0), oleic (C18:1n-9), and α-linolenic (C18:3n-3). Oleic acid was the highest, representing 48% of the total fatty acids. As previously mentioned, other minority compounds were identified and quantified, and the values obtained are shown in Table 9. In this regard, after the majority compounds, the reformulated gluten-free breads were also sources of cis-11-vaccenic acid (C18:1n-7), palmitoleic acid (C16:1n-7), cis-11-eicosenoic acid (C20:1n-9), behenic acid (C22:0), erucic acid (C22:1n-9), lignoceric acid (C24:0), and arachidic acid (C20:0). The remaining compounds were found in lower amounts (<10 mg per 100 g of gluten-free bread).

In the breads enriched with moringa leaf, a decrease in the following fatty acids was observed: myristic acid, palmitic acid, palmitoleic acid, stearic acid, trans-11-vaccenic acid, oleic acid, cis-11-vaccenic acid, linoleic acid, α-linolenic acid, arachidic acid, cis-11-eicosenoic acid, cis-11,14-eicosadienoic acid, heneicosanoic acid, behenic acid, tricosanoic acid, and lignoceric acid. This can be justified by the action of moringa and its components to metabolize fat. However, butyric acid, caproic acid, caprylic acid, capric acid, capric acid, lauric acid, pentadecanoic acid, heptadecanoic acid, elaidic acid, rumenic acid, cis-9, trans-11-linoleic acid, cis-11,14,17-eicosatrienoic acid, Erucic acid, cis-13,16-docosadienoic acid, and cis-4,7,10,13,16,19-docosahexaenoic acid showed an increase when moringa leaf was added. This was due to the high content of these fatty acids in moringa leaf [91,117].

Although the literature on this subject is scarce, similar bakery products have obtained comparable results. For example, α-linolenic, oleic, myristic, and palmitic were also the main fatty acids found in muffins enriched with moringa leaf flour in different concentrations [118]. This finding was also verified by Paramo- Calderón et al. [79], where they made tortillas enriched with moringa and found that their samples contained mostly oleic, palmitic and linoleic fatty acids authors using moringa-enriched tortillas showed similar findings with the main fatty acids being oleic, palmitic, and linoleic.

Regarding the differences found between the samples, it should be noted that with the inclusion of the moringa leaf, fatty acids decreased, but in some monounsaturated and polyunsaturated fatty acids, such as elaidic acid (9t-C18: 1), cis-9, trans-11-linoleic acid (9c,11t-C18:2 (CLA)), Cis-11,14,17-eicosatrienoic acid (C20: 3n-3), Erucic acid (C22: 1n-9), Cis-13,16-docosadienoic acid (C22:2n-6), and Cis-4,7,10,13,16,19-docosahexaenoic acid (C22:6n-3), an increase was observed with the addition of moringa leaf to gluten-free bread, highlighting AM and QM bread.

Taking into account the results obtained, the formulated bread compared could be a good source of fatty acids to commercial bread, especially the monounsaturated fatty acids and n-3 acids highlighting in the BR bread. There are other benefits, as monounsaturated and n-3 fatty acids are involved in the prevention of cardiovascular disease because they reduce blood cholesterol levels [119].

### 3.9. Folate Content

Table 10 shows the concentration of each of the four folate monoglutamates, as well as the total folate content and the content of 5-formyl-5,6,7,7,8-tetrahydrofolic acid (5FTHF), 5,6,7,8-tetrahydrofolic acid (THF), 5-methyl-5,6,7,8-tetrahydrofolic acid (5MTHF), and folic acid (FA) in the six gluten-free breads developed and one commercial gluten-free bread. In general, an increase in all folate monoglutamates was observed with moringa leaf fortification. This finding coincides with the confirmation of M. yang et al. [90], who reported that moringa leaf in bakery products increased the concentration of folic acid in cookies and bread. The total folate concentration was the highest in the AM bread (4443.58 µg folic acid equivalents/100 g FW), followed by the BRM (3804.42 µg folic acid equivalents/100 g FW), A (2952.50 µg folic acid equivalents/100 g FW), BR (2144. 76 µg folic acid equivalents/100 g FW), QM (1395.86 µg folic acid equivalents/100 g FW), and COM breads (639.96 µg folic acid equivalents/100 g FW), with significant differences of *p* < 0.01. Regarding the 5M-THF (5-methyltetrahydrofolate) content, significant differences of *p* < 0.01 were observed between the different breads, except for the QM bread compared to the Q bread. Regarding the tetrahydrofolate (THF) content, significant differences (*p* < 0.01) were also found among the different breads, except for the QM bread with BRM and A. Regarding the folic acid (FA) compound, significant differences of *p* < 0.01 were observed, except in the Q bread compared to the BR and COM bread. For the 5F-THF (5-formyltetrahydrofolate) content, significant differences were observed among all the samples; in fact, the BRM bread was found to have the highest content and the COM bread the lowest.

The main form of natural folate found to be most abundant in all of our formulated samples was 5M-THF. The main form of natural folate found to be most abundant in all of our formulated samples was 5M-THF, which is in agreement with the results of studies that recognize 5M-THF as the predominant form of folate in pseudocereals (quinoa, amaranth, and buckwheat) [120] and legumes (chickpea) [121]. According to the study by Peñalver et al. [35], the most predominant natural form of *Moringa oleifera* is THF, which may be why an increase in enriched breads was observed, as this natural form of folate does not stand out in food products [122].

According to the market consulting firm Mintel, Spain ranks third as the country in the world that sells the most gluten-free products to the market [123]. In addition, the food consumption report reported that Spain increased its consumption of gluten-free products by 3.08% in 2020, and reported that the Spanish population consumed 8.5g per day of gluten-free bread [124]. Considering these data, the consumption of the study bread could achieve 30–80% (QM bread; AM bread) RDI of folate (400 µg). Brevik et al. [125] reported that bread is one of the foods that contributes most to total folate intake, even more than other folate-containing foods such as vegetables and fruits.

The levels of the four folate monoglutamates were affected by the inclusion of moringa leaf to the bread, as a significant increase of these was observed, highlighting BRM bread by the amount of FA and 5F-THF and AM bread by the value of THF and 5M-TFH. From a nutritional point of view, BRM bread would be a great contribution to the celiac patient diet, as this diet is poor in folic acid [1].

### 3.10. Sensory Analysis

In the sensory analysis, different parameters for the organoleptic quality of each gluten-free bread were evaluated and tasters were asked to rank the samples from highest to lowest according to their taste preference. A total of 35 people participated in the analysis; 61% were women and 39% were men. The age of the participants ranged from 18–59. Furthermore, 90.3% of participants indicated that they liked the bread in the sensory analysis, with 60.9% preferring the “village bread” variety as opposed to the industrial variety frequently sold in supermarkets. In addition, 78.3% reported eating bread every day, while 13.0% reported eating bread 4–5 times a week and 8.7% 1–2 times a week. Regarding the habitual consumption of gluten-free products, 56.5% of volunteers reported having tried a gluten-free product even though they did not usually consume them, while the rest of the tasters had never tried a gluten-free product.

**Figure 3:**
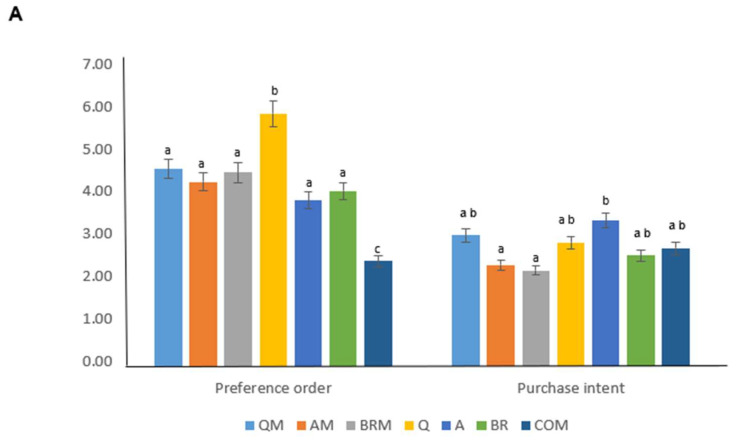

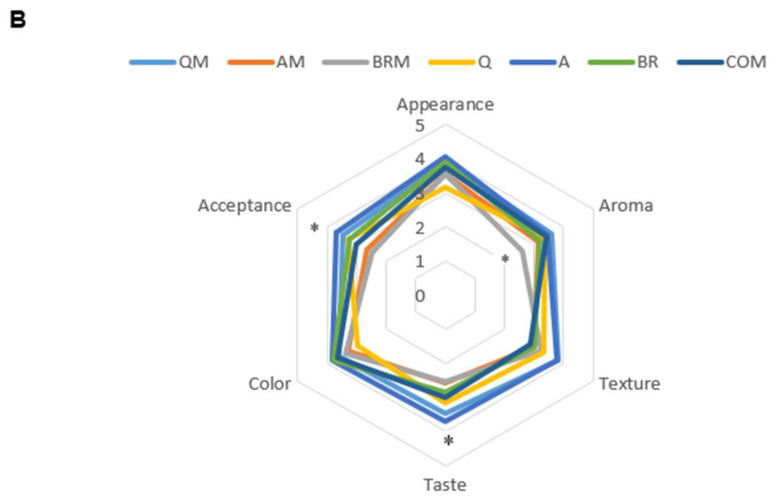
Purchase intent and preference order (**A**) and sensory analysis (**B**) of reformulated gluten-free breads. Different letters in A and * in B denote significant differences among reformulated brownies (*p* < 0.05). Q: sourdough quinoa; A: sourdough amaranth; BR: sourdough brown rice; QM: sourdough quinoa and *Moringa oleifera*; AM: sourdough amaranth and *Moringa oleifera*; BRM: sourdough amaranth and *Moringa oleifera*; COM: commercial.

The samples obtained the following order of preference from the participants: COM < A < BR < AM < QM < BRM < Q as can be seen in Figure 3A, which shows higher significant preference by participants for the Q bread obtained and the COM bread shows lower significant preference by participants. In terms of purchase intention, bread A scored significantly higher in purchase intention and BRM bread scored the lowest. Therefore, if introduced in the market, it is possible that the formulated breads would not be rejected, even though the color would be much darker than the current common gluten-free breads on the market.

In the sensory evaluation, the A bread stood out, which obtained the best qualification in appearance, flavor, color, and acceptability. However, for the attributes of aroma and texture, the QM bread obtained the highest score, as can be seen in the Figure 3B. The BRM bread stood out for its low scores in aroma (2.61), taste (2.52), and overall acceptability (2.48). These values may be due to the attributes of moringa, as brown rice is a milder cereal than the pseudocereals amaranth and quinoa, which camouflage the strong attributes of moringa, because reformulation of foods could be responsible of changes in sensory attributes [126,127,128,129].

In general, the breads enriched with moringa leaf were not accepted by the panelists, except for the aroma, appearance and texture of the QM bread. The color, taste and acceptance, higher values were found in the control breads, particularly the A bread.

## 4. Conclusions

Gluten-free bread is one of the basic products for celiac patients or people who follow a gluten-free diet. A functional and attractive bread can be obtained through the use of pseudocereal (amaranth, quinoa, and buckwheat), chickpea flour, and psyllium, with the addition of moringa leaf. Bread fortified with moringa leaf obtained the highest protein content (source of protein according to Regulation (EC) No. 1924/2006), total dietary fiber (high in fiber according to Regulation (EC) No. 1924/2006), insoluble fiber, soluble fiber, minerals, antioxidant capacity, phenolic compounds, folate content, sugar, amino acids (methionine, phenylalanine, hydroxyproline, and cysteine), and fatty acids (docosahexaenoic acid). However, considering the bioavailability of minerals (Fe, Ca, Mn, Zn, Sr, S, and B), the control breads with sourdough stood out for higher bioaccessibility of minerals. The redder and darker shades of the moringa leaf reveal encouraging results regarding the aroma and texture, but not for the acceptability of the consumers, who preferred the sensory attributes that the pseudocereal contributed to the bread without moringa.

In conclusion, our study demonstrates the significant potential for utilizing bread, a fundamental dietary staple, as a means to contribute to the reduction in some nutrient deficiencies specific to gluten-free bread. In pursuit of these objectives, our research successfully integrates moringa into gluten-free bread, resulting in a noteworthy increase in its nutritional richness, particularly regarding protein, amino acids, and essential minerals. Moreover, the incorporation of moringa heightens its capacity to contribute to improved health by augmenting the levels of soluble dietary fiber. Our findings are the first to document *Moringa oleifera*’s incorporation of this health-beneficial soluble fiber into gluten-free bread.

To the best of our knowledge, this is the first report of a gluten-free sourdough bread with a pseudocereal formulation containing moringa, a formulation that a tasters’ panel found to be acceptable; that meets the requirements to be labelled as a functional food (source of protein and high fiber) product; and that incorporates an unconventional crop that promotes health benefits, is resistant to drought and highly productive.

## Figures and Tables

**Table 1:** Gluten-free bread ingredients.

Ingredients	Control			Enriched Breads		
	Q	A	BR	QM	AM	BRM
Corn starch (g)	100	100	100	100	100	100
Salt (g)	5	5	5	5	5	5
Psyllium (g)	5	5	5	5	5	5
Sourdough (g)	50	50	50	50	50	50
Olive oil (mL)	10	10	10	10	10	10
Amaranth flour (g)	22	22	22	22	22	22
Buckwheat flour (g)	62	62	62	62	62	62
Chickpea flour (g)	42	42	42	42	42	42
Water (ml)	225	225	225	225	225	225
Sugar (g)	4	4	4	4	4	4
*Moringa oleifera* (g)	-	-	-	13.66	13.66	13.66

Q: sourdough quinoa; A: sourdough amaranth; BR: sourdough brown rice; QM: sourdough quinoa and *Moringa oleifera*; AM: sourdough amaranth and *Moringa oleifera*; BRM: sourdough amaranth and *Moringa oleifera*; COM: commercial.

**Table 2:** Chemical analyses of gluten-free breads (g/100 g).

Parameters	Sample						
	Q	A	BR	QM	AM	BRM	COM
Moisture	32.34 ± 0.07 ^b; 1^	33.46 ± 0.25 ^c; 2^	36.76 ± 0.25 ^d; 2^	40.31 ± 0.10 ^a; 1^	41.54 ± 0.12 ^a; 2^	40.20 ± 0.21 ^a; 2^	42.00 ± 0.18 ^a^
Ash	2.01 ± 0.02 ^a; 1^	1.99 ± 0.02 ^a; 1^	2.03 ± 0.05 ^a; 1^	2.33 ± 0.01 ^c; 1^	2.29 ± 0.17 ^c; 1^	2.29 ± 0.05 ^c; 1^	0.01 ± 0.01 ^b^
Total fat	1.63 ± 0.02 ^a; 1^	1.25 ± 0.08 ^b; 2^	1.16 ± 0.11 ^b; 3^	1.72 ± 0.01 ^a; 1^	1.71 ± 0.01 ^a; 2^	1.32 ± 0.03 ^b; 3^	3.25 ± 0.11 ^c^
Proteins	5.43 ± 0.02 ^a; 1^	5.40 ± 0.01 ^a; 2^	5.25 ± 0.03 ^a; 3^	6.76 ± 0.10 ^b; 1^	6.48 ± 0.03 ^c; 2^	5.77 ± 0.11 ^d; 3^	2.96 ± 0.10 ^e^
Total fiber	10.77 ± 0.04 ^b; 1^	8.98 ± 0.57 ^c; 2^	8.41 ± 0.23 ^c; 3^	11.83 ± 0.31 ^a; 1^	11.70 ± 0.23 ^a; 2^	11.33 ± 0.12 ^a, b; 3^	3.09 ± 0.03 ^d^
Soluble fiber	2.58 ± 0.32 ^a, b; 1^	2.19 ± 0.46 ^b; 1^	1.40 ± 0.21 ^c; 2^	3.16 ± 0.02 ^a; 1^	3.12 ± 0.06 ^a; 1^	2.99 ± 0.03 ^a; 2^	1.06 ± 0.08 ^c^
Insoluble fiber	8.19 ± 0.36 ^a; 2^	6.80 ± 0.10 ^b; 1^	7.01 ± 0.02 ^b; 1^	8.67 ± 0.29 ^a; 2^	8.58 ± 0.28 ^a; 1^	8.34 ± 0.15 ^a; 1^	3.09 ± 0.03 ^c^
Carbohydrates	47.82 ± 0.06 ^c; 1^	48.92 ± 1.33 ^d; 1^	46.38 ± 0.20 ^c; 1^	37.06 ± 0.15 ^a, b; 1^	36.27 ± 0.09 ^b; 1^	39.09 ± 0.17 ^a; 1^	45.24 ± 0.29 ^c^

Q: sourdough quinoa; A: sourdough amaranth; BR: sourdough brown rice; QM: sourdough quinoa and *Moringa oleifera*; AM: sourdough amaranth and *Moringa oleifera*; BRM: sourdough amaranth and *Moringa oleifera*; COM: commercial. Different subscript letters indicate significant differences (*p* < 0.005) between columns ^a–e^. Different subscripts indicate differences between samples depending on the sourdough ^1–3^.

**Table 3:** pH, TTA, and acid contents in the gluten-free breads.

Sample	pH	TTA (ml NaOH/10 g)	L-Lactic Acid (g/kg)	Acetic Acid (g/kg)
Q	6.14 ± 0.07 ^d; 1^	6.20 ± 0.10 ^b, c; 1^	0.19 ± 0.06 ^a, e; 1^	1.30 ± 0.01 ^c; 1^
A	5.75 ± 0.08 ^c; 2^	6.40 ± 0.30 ^c; 1^	0.47 ± 0.05 ^c; 2^	1.11 ± 0.02 ^b; 2^
BR	6.16 ± 0.08 ^d; 3^	5.60 ± 0.40 ^b; 2^	0.23 ± 0.03 ^e; 3^	1.18 ± 0.01 ^a; 3^
QM	5.45 ± 0.04 ^a; 1^	7.42 ± 0.10 ^a; 1^	0.23 ± 0.06 ^a, e; 1^	1.34 ± 0.01 ^d; 1^
AM	5.25 ± 0.04 ^b; 2^	7.55 ± 0.13 ^a; 1^	0.63 ± 0.04 ^b; 2^	1.18 ± 0.02 ^a; 2^
BRM	5.65 ± 0.05 ^c; 3^	7.20 ± 0.10 ^a; 2^	0.37 ± 0.06 ^c, a, e; 3^	1.26 ± 0.01 ^e; 3^
COM	4.89 ± 0.08 ^e^	4.37 ± 0.30 ^d^	0.17 ± 0.02 ^d, a, e^	0.35 ± 0.01 ^f^

TTA: total titratable acidity; Q: sourdough quinoa; A: sourdough amaranth; BR: sourdough brown rice; QM: sourdough quinoa and *Moringa oleifera*; AM: sourdough amaranth and *Moringa oleifera*; BRM: sourdough amaranth and *Moringa oleifera*; COM: commercial. Different subscript letters indicate significant differences (*p* < 0.005) between rows ^a–f^. Different subscripts indicate differences between samples depending on the sourdough ^1–3^.

**Table 4:** Colorimetric parameters for the different bread samples. Results are expressed as mean ± SD.

Sample	L*	C*	h*	a*	b*
Q	67.61 ± 4.26 ^b^ *	19.26 ± 2.67 ^a^	86.35 ± 1.60 ^b^	1.17 ± 0.35 ^b^ *	19.22 ± 2.71 ^a^
A	67.87 ± 0.76 ^b^	19.63 ± 1.67 ^a^	87.59 ± 1.58 ^b^	0.80 ± 0.44 ^b^	19.61 ± 1.70 ^a^
BR	71.86 ± 1.54 ^b^ *	20.23 ± 3.73 ^a^	88.66 ± 1.82 ^b^	0.40 ± 0.51 ^b^ *	20.22 ± 3.75 ^a^
QM	50.91 ± 0.16 ^a^ *	19.76 ± 0.27 ^a^	80.92 ± 0.09 ^a^	3.12 ± 0.03 ^a^ *	19.42 ± 0.03 ^a^
AM	53.68 ± 0.07 ^a^	17.83 ± 0.19 ^a^	81.17 ± 0.07 ^a^	2.77 ± 0.03 ^a^	17.53 ± 0.02 ^a^
BRM	53.35 ± 0.04 ^a^ *	15.97 ± 0.06 ^a^	80.65 ± 0.07 ^a^	2.63 ± 0.02 ^a^ *	15.79 ± 0.03 ^a^
COM	84.39 ± 1.49 ^c^	18.71 ± 0.59 ^a^	91.29 ± 0.91 ^c^	0.22 ± 0.10 ^c^	18.59 ± 0.61 ^a^

Q: sourdough quinoa; A: sourdough amaranth; BR: sourdough brown rice; QM: sourdough quinoa and *Moringa oleifera*; AM: sourdough amaranth and *Moringa oleifera*; BRM: sourdough amaranth and *Moringa oleifera*; COM: commercial. Different subscript letters indicate significant differences (*p* < 0.005) between rows ^a–c^. *: Indicates the differences between samples depending on the sourdough.

**Table 5:** Total phenolic content (TPC) (mg GAE g-1) and antioxidant activity (µmol TE g-1) of the gluten-free breads.

Sample	TPC	Antioxidant Activity			
		ABTS	DPPH	FRAP	ORAC
Q	7.22 ± 1.82 ^b; 1^	39.81 ± 0.10 ^d; 1^	16.81 ± 0.84 ^b; 1^	4.52 ± 0.14 ^c; 1^	880.93 ± 32.47 ^c; 1^
A	6.44 ± 0.83 ^b; 2^	41.03 ± 0.30 ^c; 2^	16.73 ± 0.33 ^b; 1^	6.87 ± 0.09 ^a; 2^	959.95 ± 40.02 ^d; 2^
BR	5.65 ± 0.86 ^b d; 3^	47.39 ± 0.05 ^b; 3^	15.36 ± 0.10 ^c; 2^	5.19 ± 0.23 ^b; 3^	735.82 ± 4.67 ^e; 3^
QM	11.88 ± 0.11 ^a; 1^	50.78 ± 0.26 ^a; 1^	25.82 ± 0.21 ^a; 1^	6.33 ± 0.19 ^a; 1^	1192.82 ± 0.74 ^a b; 1^
AM	15.61 ± 0.60 ^c; 2^	50.95 ± 0.24 ^a; 2^	26.96 ± 0.11 ^d; 1^	9.37 ± 0.40 ^d; 2^	1235.52 ± 0.44 ^b; 2^
BRM	9.67 ± 0.10 ^a; 3^	50.77 ± 0.19 ^a; 3^	25.34 ± 0.10 ^a; 2^	6.93 ± 0.05 ^a; 3^	1170.65 ± 0.52 ^a; 3^
COM	3.72 ± 0.03 ^d^	28.19 ± 0.22 ^e^	6.39 ± 0.16 ^e^	1.34 ± 0.11 ^e^	463.25 ± 0.91 ^f^

Q: sourdough quinoa; A: sourdough amaranth; BR: sourdough brown rice; QM: sourdough quinoa and *Moringa oleifera*; AM: sourdough amaranth and *Moringa oleifera*; BRM: sourdough amaranth and *Moringa oleifera*; COM: commercial. Different subscript letters indicate significant differences (*p* < 0.005) between rows ^a–f^. Different subscripts indicate differences between samples depending on the sourdough ^1–3^.

**Table 6:** Fructose, glucose, maltose, and sucrose content (g/100 g) of gluten-free breads.

Sample	Sucrose	Fructose	Glucose	Maltose
Q	2.10 ± 0.03 ^a; 1^	0.06 ± 0.01 ^a; 1^	0.13 ± 0.01 ^a; 1^	1.05 ± 0.01 ^a; 1^
A	1.16 ± 0.01 ^b; 2^	0.06 ± 0.01 ^a; 1^	0.06 ± 0.01 ^b; 2^	0.15 ± 0.02 ^e; 2^
BR	1.11 ± 0.01 ^b; 3^	0.02 ± 0.01 ^b; 2^	0.02 ± 0.01 ^c; 3^	0.06 ± 0.01 ^d; 3^
QM	2.56 ± 0.02 ^d; 1^	0.15 ± 0.00 ^c; 1^	0.24 ± 0.02 ^d; 1^	1.01 ± 0.02 ^b; 1^
AM	1.48 ± 0.01 ^e; 2^	0.15 ± 0.01 ^c; 1^	0.18 ± 0.01 ^e; 2^	0.15 ± 0.01 ^e; 2^
BRM	1.29 ± 0.01 ^f; 3^	0.11 ± 0.02 ^c; 2^	0.12 ± 0.01 ^c; 3^	0.05 ± 0.02 ^d; 3^
COM	0.11 ± 0.01 ^c^	0.03 ± 0.01 ^b^	0.78 ± 0.02 ^f^	2.74 ± 0.08 ^c^

Q: sourdough quinoa; A: sourdough amaranth; BR: sourdough brown rice; QM: sourdough quinoa and *Moringa oleifera*; AM: sourdough amaranth and *Moringa oleifera*; BRM sourdough amaranth and *Moringa oleifera*; COM: commercial. Different subscript letters indicate significant differences (*p* < 0.005) between rows ^a−f^. Different subscripts indicate differences between samples depending on the sourdough ^1−3^.

**Table 7:** Mineral contents of gluten-free breads.

Mineral	Sample						
	QM	AM	BRM	Q	A	BR	COM
B (mg/Kg)	1.37 ± 0.12 ^a; 1^	2.28 ± 0.34 ^c; 2^	0.99 ± 0.34 ^a; 3^	0.10 ± 0.02 ^b; 1^	0.16 ± 0.03 ^b; 2^	0.12 ± 0.03 ^b; 3^	nd
Ca (g/100 g)	0.41 ± 0.01 ^a b; 1^	0.46 ± 0.05 ^b; 2^	0.38 ± 0.01 ^a c; 3^	0.29 ± 0.02 ^d; 1^	0.33 ± 0.01 ^c d; 2^	0.30 ± 0.02 ^d; 3^	0.11 ± 0.01 ^e^
Cu (mg/Kg)	1.08 ± 0.05 ^d; 1^	0.34 ± 0.01 ^a c; 2^	0.32 ± 0.01 ^a c; 3^	0.93 ± 0.07 ^d; 1^	0.16 ± 0.01 ^a b; 2^	0.16 ± 0.01 ^b; 3^	0.31 ± 0.06 ^c^
Fe (mg/Kg)	5.44 ± 0.19 ^a c; 1^	7.54 ± 0.42 ^d; 2^	4.66 ± 0.04 ^b c; 3^	4.63 ± 0.35 ^c; 1^	5.91 ± 0.29 ^a; 2^	4.22 ± 0.62 ^b c; 3^	3.83 ± 0.62 ^b c^
K (g/100 g)	0.64 ± 0.02 ^a; 1^	0.54 ± 0.01 ^a b; 2^	0.46 ± 0.02 ^b; 3^	0.49 ± 0.08 ^a b; 1^	0.40 ± 0.06 ^b; 2^	0.49 ± 0.05 ^a b; 3^	0.31 ± 0.08 ^c^
Mg (g/100 g)	0.03 ± 0.01 ^a; 1^	0.04 ± 0.01 ^a; 1^	0.03 ± 0.01 ^a; 1^	0.03 ± 0.01 ^a; 1^	0.03 ± 0.01 ^a; 1^	0.03 ± 0.01 ^a; 1^	0.01 ± 0.01 ^b^
Mn (mg/Kg)	5.11 ± 0.32 ^a c; 1^	5.53 ± 0.29 ^a; 1^	4.37 ± 1.18 ^a c; 1^	3.98 ± 0.35 ^c; 1^	4.59 ± 0.26 ^a c; 1^	4.33 ± 0.12 ^a c; 1^	1.81 ± 0.25 ^b^
Pb (mg/Kg)	0.11 ± 0.04 ^a; 1^	0.08 ± 0.02 ^a b; 2^	0.07 ± 0.02 ^a b; 3^	0.10 ± 0.02 ^a b; 1^	0.06 ± 0.01 ^a b; 2^	0.06 ± 0.01 ^a b; 3^	0.05 ± 0.01 ^b^
Si (mg/kg)	20.39 ± 3.69 ^a; 1^	43.95 ± 2.14 ^b; 1^	16.50 ± 0.56 ^a; 1^	16.37 ± 2.29 ^a; 1^	26.09 ± 11.64 ^a; 1^	11.77 ± 0.66 ^a; 1^	95.91 ± 6.13 ^c^
Sr (mg/Kg)	1.35 ± 0.04 ^b; 1^	1.89 ± 0.03 ^c; 1^	1.13 ± 0.03 ^a; 1^	1.13 ± 0.06 ^a; 1^	1.64 ± 0.01 ^d; 1^	1.03 ± 0.03 ^e; 1^	0.27 ± 0.03 ^f^
S (g/100 g)	0.06 ± 0.01 ^a; 1^	0.05 ± 0.01 ^a b; 1^	0.04 ± 0.01 ^b; 1^	0.01 ± 0.01 ^c; 1^	0.01 ± 0.02 ^c; 1^	0.01 ± 0.01 ^c; 1^	0.05 ± 0.01 ^a b^
Na (g/100 g)	0.48 ± 0.02 ^a; 1^	0.46 ± 0.02 ^a; 1^	0.45 ± 0.01 ^a b; 1^	0.27 ± 0.03 ^c; 1^	0.31 ± 0.05 ^c; 1^	0.36 ± 0.01 ^b c; 1^	0.35 ± 0.03 ^c^
Ni (mg/kg)	0.21 ± 0.02 ^a c; 1^	0.24 ± 0.01 ^a; 1^	0.25 ± 0.01 ^a; 1^	0.21 ± 0.01 ^a c; 1^	0.16 ± 0.01 ^c; 1^	0.21 ± 0.03 ^a c; 1^	0.11 ± 0.01 ^b^
Zn (mg/Kg)	7.24 ± 0.11 ^a; 1^	6.77 ± 0.21 ^b; 2^	6.35 ± 0.30 ^b; 3^	6.66 ± 0.10 ^a b; 1^	5.70 ± 0.08 ^c; 2^	5.19 ± 0.05 ^d; 3^	3.70 ± 0.05 ^e^

Q: gluten-free bread with sourdough quinoa; A: gluten-free bread with sourdough amaranth; BR: gluten-free bread with sourdough brown rice; QM: gluten-free bread with sourdough quinoa and *Moringa oleifera*; AM: gluten-free bread with sourdough amaranth and *Moringa oleifera*; BRM: Gluten-free bread with sourdough amaranth and *Moringa oleifera*; COM: commercial gluten-free bread. Nd: not identified. Different subscript letters indicate significant differences (*p* < 0.005) between columns ^a−f^. Different subscripts indicate differences between samples depending on the sourdough ^1−3^.

**Table 8:** Amino acid profiles of the gluten-free bread (mean ± standard deviation values).

Amino Acids (mg/100 g)	Samples						
	QM	AM	BRM	Q	A	BR	COM
Essential amino acids							
Histidine	131.26 ± 9.02 ^a; 1^	108.80 ± 14.02 ^a; 1^	104.14 ± 17.34 ^a; 1^	239.64 ± 32.91 ^b; 1^	233.36 ± 45.10 ^b; 1^	239.79 ± 6.36 ^b; 1^	121.06 ± 7.24 ^a^
Arginine	455.19 ± 39.56 ^a b; 1^	366.18 ± 56.67 ^a; 1^	398.37 ± 59.32 ^a; 2^	557.89 ± 22.24 ^b; 1^	633.71 ± 29.70 ^b; 1^	758.92 ± 44.01 ^c; 2^	412.19 ± 22.19 ^a^
Threonine	197.03 ± 16.58 ^a; 1^	159.64 ± 21.04 ^a; 1^	155.09 ± 21.55 ^a; 1^	330.23 ± 32.65 ^b; 1^	317.05 ± 69.02 ^b; 1^	347.75 ± 13.06 ^b^	173.89 ± 11.43 ^a^
Valine	198.76 ± 16.70 ^a; 1^	142.75 ± 25.41 ^a; 2^	149.60 ± 19.00 ^a; 1^	423.28 ± 12.04 ^b; 1^	357.38 ± 35.37 ^c; 2^	432.27 ± 9.97 ^b; 1^	168.37 ± 16.83 ^a^
Methionine	72.42 ± 2.30 ^a; 1^	77.68 ± 5.00 ^a; 1^	55.85 ± 38.85 ^a; 1^	58.19 ± 19.48 ^a; 1^	56.90 ± 10.70 ^a; 1^	54.24 ± 2.11 ^a; 1^	66.79 ± 4.40 ^a^
Lysine	267.46 ± 29.00 ^a; 1^	215.39 ± 21.84 ^a; 2^	223.56 ± 8.08 ^a; 1^	414.43 ± 6.76 ^b; 1^	346.05 ± 17.67 ^c; 2^	440.70 ± 38.32 ^b; 1^	252.63 ± 31.58 ^a^
Isoleucine	184.09 ± 36.70 ^a; 1^	113.57 ± 24.81 ^b; 2^	127.05 ± 24.92 ^b; 1 2^	365.94 ± 11.47 ^c; 1^	301.75 ± 12.12 ^c; 1 2^	354.96 ± 27.05 ^c; 2^	144.79 ± 27.26 ^a b^
Leucine	322.62 ± 31.53 ^a b c; 1^	247.41 ± 58.11 ^b; 2^	245.13 ± 22.35 ^a b; 1 2^	415.92 ± 10.81 ^c; 1^	360.54 ± 6.28 ^a c; 2^	439.29 ± 56.49 ^c; 1 2^	270.64 ± 26.52 ^a b^
Phenylalanine	553.40 ± 46.84 ^a; 1^	442.41 ± 20.07 ^c; 2^	543.54 ± 14.46 ^a; 1 2^	249.32 ± 20.33 ^b; 1^	203.27 ± 32.62 ^b; 2^	197.06 ± 26.26 ^b; 1 2^	213.73 ± 6.76 ^b^
Total EAA	2372.37 ± 178.66 ^a c; 1^	1873.83 ± 211.66 ^b c; 2^	1994.18 ± 204.14 ^c; 1^	3055.07 ± 109.31 ^d; 1^	2810.00 ± 205.23 ^d; 2^	3264.99 ± 146.97 ^d; 1^	1824.08 ± 150.28 ^b c^
Non-essential amino acids							
Serine	262.76 ± 25.86 ^a; 1^	232.54 ± 16.18 ^a; 1^	217.63 ± 31.07 ^a; 1^	383.80 ± 10.85 ^b; 1^	348.77 ± 38.72 ^b; 1^	393.80 ± 39.38 ^b; 1^	230.83 ± 24.41 ^a^
Asparagine	516.11 ± 52.79 ^a b; 1^	397.68 ± 53.98 ^b; 2^	414.13 ± 41.09 ^b; 2^	757.94 ± 35.79 ^c; 1^	638.76 ± 46.39 ^a c; 2^	658.72 ± 14.69 ^c; 2^	446.27 ± 59.08 ^b^
Glutamic acid	824.61 ± 72.28 ^a b; 1^	651.26 ± 97.66 ^a; 1^	688.90 ± 68.48 ^a; 1^	1012.19 ± 26.53 ^b; 1^	984.45 ± 67.31 ^b; 1^	1207.97 ± 136.12 ^c b; 1^	709.62 ± 115.04 ^a^
Glycine	280.00 ± 25.10 ^a b; 1^	241.38 ± 30.31 ^b; 1^	224.60 ± 32.35 ^b; 1^	361.85 ± 25.88 ^a; 1^	356.23 ± 48.72 ^a; 1^	361.20 ± 16.73 ^a; 1^	254.60 ± 23.82 ^b^
Alanine	111.00 ± 10.98 ^a d; 1^	50.02 ± 30.43 ^d; 2^	99.16 ± 33.59 ^a d; 3^	449.83 ± 19.59 ^b; 1^	323.24 ± 9.49 ^c; 2^	354.37 ± 26.57 ^c; 3^	122.35 ± 20.83 ^a^
Proline	278.46 ± 35.13 ^a; 1^	192.14 ± 14.01 ^a; 2^	171.99 ± 24.69 ^a; 2^	555.71 ± 63.13 ^b; 1^	491.28 ± 65.74 ^b; 2^	521.55 ± 20.42 ^b; 2^	240.00 ± 25.32 ^a^
Cysteine	83.43 ± 8.06 ^a; 1^	133.74 ± 29.61 ^b; 2^	137.40 ± 22.39 ^b; 2^	59.12 ± 7.16 ^a; 1^	75.05 ± 12.52 ^a; 2^	63.18 ± 1.01 ^a; 2^	69.04 ± 6.73 ^a^
Tyrosine	87.92 ± 9.13 ^a; 1 2^	62.02 ± 14.31 ^a; 2^	71.01 ± 11.47 ^a; 1^	182.36 ± 20.37 ^b; 1 2^	194.14 ± 10.17 ^b c; 2^	236.45 ± 25.07 ^c; 1^	74.56 ± 8.42 ^a^
Hydroxyproline	5.08 ± 2.02 ^a; 1^	2.83 ± 1.98 ^a b; 1^	4.06 ± 0.64 ^a c; 1^	1.77 ± 0.10 ^b c; 1^	1.83 ± 1.01 ^b c; 1^	2.46 ± 0.12 ^a c; 1^	0.26 ± 0.09 ^b^
Taurine	nd	nd	nd	nd	nd	nd	nd
Total NEAA	2449.37 ± 224.53 ^a; 1^	1963.60 ± 252.36 ^a; 2^	2028.88 ± 236.29 ^a; 1 2^	3764.57 ± 98.07 ^b; 1^	3413.75 ± 280.92 ^b; 2^	3799.70 ± 279.34 ^b; 1 2^	2147.54 ± 235.50 ^a^
Total AA	4821.74 ± 399.53 ^a; 1^	3837.43 ± 463.93 ^a; 2^	4023.06 ± 439.94 ^a; 1 2^	6819.65 ± 204.87 ^b; 1^	6223.76 ± 484.31 ^b; 2^	7064.69 ± 420.17 ^b; 1 2^	3971.62 ± 383.11 ^a^

Q: sourdough quinoa; A: sourdough amaranth; BR: sourdough brown rice; QM: sourdough quinoa and *Moringa oleifera*; AM: sourdough amaranth and *Moringa oleifera*; BRM: sourdough amaranth and *Moringa oleifera*; COM: commercial. Different subscript letters indicate significant differences (*p* < 0.005) between columns ^a–d^. NEAA: non-essential amino acids; AA: essential amino acids. Nd: not identified. Different subscripts indicate differences between samples depending on the sourdough ^1–3^.

**Table 9:** Fatty acid profiles (mg 100 g^−1^) of the gluten-free breads.

Fatty Acids	Sample						
	QM	AM	BRM	Q	A	BR	COM
C4:0	2.85 ± 0.25 ^a; 1^	3.60 ± 0.38 ^b; 2^	1.91 ± 0.02 ^c; 3^	0.00 ± 0.00 ^d; 1^	0.00 ± 0.00 ^d; 2^	0.00 ± 0.00 ^d; 3^	2.49 ± 0.36 ^a c^
C6:0	1.64 ± 0.14 ^a; 1^	2.18 ± 0.28 ^b; 2^	1.14 ± 0.01 ^c d; 1^	0.73 ± 0.10 ^d; 1^	0.99 ± 0.03 ^c d; 2^	0.85 ± 0.20 ^c d; 1^	1.41 ± 0.24 ^a c^
C8:0	1.63 ± 0.15 ^a b; 1^	2.01 ± 0.20 ^b; 2^	1.12 ± 0.03 ^c; 1^	0.84 ± 0.11 ^c; 1^	1.28 ± 0.16 ^a c; 2^	1.07 ± 0.24 ^c; 1^	11.23 ± 0.24 ^d^
C10:0	1.07 ± 0.11 ^a b; 1 2^	1.40 ± 0.18 ^b; 2^	0.73 ± 0.03 ^a c; 1^	0.29 ± 0.26 ^c; 1 2^	0.36 ± 0.31 ^c; 2^	0.26 ± 0.22 ^c; 1^	8.17 ± 0.24 ^d^
C11:0	0.00 ± 0.00 ^a; 1^	0.00 ± 0.00 ^a; 1^	0.00 ± 0.00 ^a; 1^	0.00 ± 0.00 ^a; 1^	0.00 ± 0.00 ^a; 1^	0.00 ± 0.00 ^a; 1^	0.00 ± 0.00 ^a^
C12:0	0.88 ± 0.07 ^a; 1^	1.08 ± 0.12 ^a; 2^	0.62 ± 0.03 ^b; 3^	0.40 ± 0.04 ^c; 1^	0.49 ± 0.09 ^b c; 2^	0.39 ± 0.04 ^c; 3^	0.90 ± 0.09 ^a^
C13:0	0.00 ± 0.00 ^a; 1^	0.00 ± 0.00 ^a; 1^	0.00 ± 0.00 ^a; 1^	0.00 ± 0.00 ^a; 1^	0.00 ± 0.00 ^a; 1^	0.00 ± 0.00 ^a; 1^	0.00 ± 0.00 ^a^
C14:0	3.14 ± 0.09 ^a b; 1 2^	3.42 ± 0.15 ^b; 2^	2.50 ± 0.08 ^c; 1^	3.12 ± 0.21 ^a b; 1 2^	3.00 ± 0.06 ^a; 2^	3.47 ± 0.03 ^a b; 1^	3.35 ± 0.19 ^a b^
C14:1n-5	0.00 ± 0.00 ^a; 1^	0.00 ± 0.00 ^a; 1^	0.00 ± 0.00 ^a; 1^	0.00 ± 0.00 ^a; 1^	0.00 ± 0.00 ^a; 1^	0.00 ± 0.00 ^a; 1^	0.00 ± 0.00 ^a^
C15:0	1.64 ± 0.04 ^a; 1^	1.73 ± 0.05 ^a; 1^	1.16 ± 0.02 ^b; 2^	1.56 ± 0.15 ^a; 1^	1.53 ± 0.04 ^a b; 1^	1.42 ± 0.32 ^a b; 2^	0.75 ± 0.02 ^c^
C15:1n-5	0.00 ± 0.00 ^a; 1^	0.00 ± 0.00 ^a; 1^	0.00 ± 0.00 ^a; 1^	0.00 ± 0.00 ^a; 1^	0.00 ± 0.00 ^a; 1^	0.00 ± 0.00 ^a; 1^	0.00 ± 0.00 ^a^
C16:0	373.82 ± 11.24 ^a; 1^	482.83 ± 0.93 ^b c; 2^	326.91 ± 16.05 ^c; 1^	473.07 ± 28.14 ^c; 1^	510.11 ± 16.91 ^c; 2^	556.47 ± 6.23 ^d; 1^	195.95 ± 16.87 ^e^
C16:1n-7	20.18 ± 0.47 ^a; 1^	27.80 ± 0.21 ^b e; 2^	17.31 ± 0.65 ^a; 1^	24.30 ± 1.07 ^c; 1^	28.17 ± 2.70 ^e; 2^	30.49 ± 0.58 ^e; 1^	3.57 ± 0.27 ^f^
C17:0	2.24 ± 0.02 ^a b; 1^	2.83 ± 0.06 ^b; 2^	1.90 ± 0.15 ^c a; 1^	1.61 ± 0.04 ^c a; 1^	1.84 ± 0.55 ^c a; 2^	1.80 ± 0.25 ^c a; 1^	1.55 ± 0.09 ^c^
C17:1n-7	0.00 ± 0.00 ^a; 1^	0.00 ± 0.00 ^a; 1^	0.00 ± 0.00 ^a; 1^	0.00 ± 0.00 ^a; 1^	0.00 ± 0.00 ^a; 1^	0.00 ± 0.00 ^a; 1^	0.00 ± 0.00 ^a^
C18:0	61.29 ± 1.13 ^a; 1^	89.39 ± 0.46 ^b; 2^	66.47 ± 1.82 ^a; 2^	124.94 ± 8.76 ^c; 1^	124.39 ± 1.11 ^c; 2^	143.56 ± 1.32 ^d; 2^	90.40 ± 5.58 ^b^
9t-C18:1	1.33 ± 0.04 ^a; 1^	1.68 ± 0.16 ^b; 2^	0.98 ± 0.02 ^c; 3^	0.00 ± 0.00 ^a; 1 2 3^	0.00 ± 0.00 ^a; 1 2 3^	0.00 ± 0.00 ^a; 1 2 3^	1.21 ± 0.11 ^a^
11t-C18:1	0.00 ± 0.00 ^a; 1 2^	0.00 ± 0.00 ^a; 1 2^	0.00 ± 0.00 ^a; 1 2^	3.92 ± 0.64 ^b; 1^	6.06 ± 1.27 ^d; 2^	4.59 ± 0.55 ^b d; 1 2^	0.00 ± 0.00 ^a^
C18:1n-9	1211.26 ± 21.30 ^e; 1^	1585.44 ± 5.65 ^a; 2^	1020.77 ± 28.00 ^b; 1^	1676.39 ± 107.74 ^a c; 1^	1841.46 ± 99.77 ^c; 2^	2045.27 ± 12.67 ^d; 1^	909.10 ± 55.57 ^b^
C18:1n-7	52.51 ± 1.22 ^a; 1^	68.43 ± 0.16 ^b; 2^	42.28 ± 0.98 ^c; 1^	56.37 ± 4.24 ^a d; 1^	59.97 ± 4.26 ^d; 2^	67.94 ± 3.04 ^b; 1^	19.89 ± 1.04 ^e^
C18:2n-6	726.22 ± 11.37 ^a b, 1^	777.80 ± 4.31 ^b c; 1^	605.49 ± 24.46 ^a; 1^	888.88 ± 63.75 ^c d; 1^	849.78 ± 13.11 ^a c d; 1^	937.65 ± 13.33 ^d; 1^	1439.02 ± 109.71 ^e^
C18:3n-6	0.46 ± 0.03 ^a; 1^	0.00 ± 0.00 ^b; 2^	0.00 ± 0.00 ^b; 2^	0.00 ± 0.00 ^b; 1 2^	0.00 ± 0.00 ^b; 1 2^	0.00 ± 0.00 ^b; 1 2^	0.00 ± 0.00 ^b^
C18:3n-3	47.22 ± 0.52 ^a b; 1^	41.92 ± 0.20 ^b c; 1^	32.53 ± 0.99 ^c; 2^	52.62 ± 4.56 ^a b; 1^	48.84 ± 9.64 ^a b; 1^	52.93 ± 1.94 ^a b; 2^	17.75 ± 0.91 ^d^
9t,11t-C18:2 (CLA)	4.57 ± 0.37 ^a b; 1^	6.02 ± 0.74 ^b; 2^	3.09 ± 0.26 ^c d; 1^	1.67 ± 0.33 ^d; 1^	2.50 ± 0.45 ^d; 2^	2.45 ± 0.57 ^a d; 1^	4.47 ± 0.79 ^c^
C20:0	15.79 ± 0.30 ^a; 1^	20.29 ± 0.09 ^e; 2^	16.36 ± 0.59 ^a; 1^	26.90 ± 2.10 ^b; 1^	30.64 ± 0.65 ^c; 2^	29.26 ± 0.98 ^b c; 1^	7.96 ± 0.22 ^d^
C20:1n-9	16.45 ± 0.29 ^a; 1^	14.89 ± 0.31 ^a; 1 2^	11.26 ± 0.36 ^b; 2^	18.10 ± 2.35 ^a; 1^	16.83 ± 1.35 ^a; 1 2^	18.04 ± 1.78 ^a; 2^	6.18 ± 0.25 ^c^
C20:2n-6	1.56 ± 0.06 ^a b; 1^	1.49 ± 0.11 ^a b; 1^	1.04 ± 0.04 ^b d; 1^	1.81 ± 0.24 ^a; 1^	2.04 ± 0.36 ^a; 1^	1.88 ± 0.33 ^a; 1^	0.68 ± 0.03 ^d^
C21:0	1.10 ± 0.05 ^a b c; 1^	1.40 ± 0.05 ^b d; 2^	0.91 ± 0.04 ^c; 1^	1.42 ± 0.44 ^b d; 1^	1.72 ± 0.12 ^d; 2^	1.50 ± 0.03 ^a b d; 1^	0.61 ± 0.08 ^c^
C20:3n-6	0.00 ± 0.00 ^a; 1^	0.00 ± 0.00 ^a; 1^	0.00 ± 0.00 ^a; 1^	0.00 ± 0.00 ^a; 1^	0.00 ± 0.00 ^a; 1^	0.00 ± 0.00 ^a; 1^	0.00 ± 0.00 ^a^
C20:4n-6	0.00 ± 0.00 ^a; 1^	0.00 ± 0.00 ^a; 1^	0.00 ± 0.00 ^a; 1^	0.00 ± 0.00 ^a; 1^	0.00 ± 0.00 ^a; 1^	0.00 ± 0.00 ^a; 1^	0.00 ± 0.00 ^a^
C20:3n-3	0.98 ± 0.03 ^a; 1^	0.73 ± 0.03 ^b; 2^	0.41 ± 0.01 ^c; 3^	0.00 ± 0.00 ^d; 1 2 3^	0.00 ± 0.00 ^d; 1 2 3^	0.00 ± 0.00 ^d; 12 3^	0.39 ± 0.37
C22:0	10.49 ± 0.20 ^a; 1^	10.91 ± 0.17 ^a; 1^	8.73 ± 0.37 ^a; 1^	15.58 ± 1.10 ^b; 1^	15.11 ± 1.99 ^b; 1^	16.35 ± 0.32 ^b; 1^	20.27 ± 0.72 ^c^
C20:5n-3	0.00 ± 0.00 ^a; 1^	0.00 ± 0.00 ^a; 1^	0.00 ± 0.00 ^a; 1^	0.00 ± 0.00 ^a; 1^	0.00 ± 0.00 ^a; 1^	0.00 ± 0.00 ^a; 1^	0.00 ± 0.00 ^a^
C22:1n-9	6.90 ± 0.23 ^a; 1^	4.35 ± 0.04 ^b; 2^	3.81 ± 0.28 ^b c; 3^	3.58 ± 0.36 ^c; 1^	1.82 ± 0.14 ^d; 2^	1.55 ± 0.35 ^d; 3^	3.61 ± 0.32 ^b c^
C22:2n-6	0.80 ± 0.18 ^b; 1^	0.76 ± 0.22 ^b; 1^	0.38 ± 0.03 ^c; 2^	0.00 ± 0.00 ^a; 1 2 3^	0.00 ± 0.00 ^a; 1 2 3^	0.00 ± 0.00 ^a; 1 2 3^	0.00 ± 0.00 ^a^
C23:0	2.00 ± 0.08 ^a b; 1^	2.36 ± 0.05 ^a; 2^	1.55 ± 0.06 ^b; 1^	3.10 ± 0.34 ^c; 1^	3.42 ± 0.22 ^c; 2^	3.19 ± 0.16 ^c; 1^	1.16 ± 0.04 ^b^
C24:0	7.17 ± 0.25 ^a; 1^	7.67 ± 0.13 ^a; 1^	5.92 ± 0.21 ^a; 1^	11.06 ± 1.31 ^b; 1^	11.21 ± 0.84 ^b; 1^	12.44 ± 1.24 ^b; 1^	7.25 ± 0.08 ^a^
C22:5n-3	0.00 ± 0.00 ^a; 1^	0.00 ± 0.00 ^a; 1^	0.00 ± 0.00 ^a; 1^	0.00 ± 0.00 ^a; 1^	0.00 ± 0.00 ^a; 1^	0.00 ± 0.00 ^a; 1^	0.00 ± 0.00 ^a^
C24:1n-9	0.00 ± 0.00 ^a; 1^	0.00 ± 0.00 ^a; 1^	0.00 ± 0.00 ^a; 1^	0.00 ± 0.00 ^a; 1^	0.00 ± 0.00 ^a; 1^	0.00 ± 0.00 ^a; 1^	0.00 ± 0.00 ^a^
C22:6n-3	1.18 ± 0.12 ^a; 1^	1.08 ± 0.16 ^a; 1^	0.56 ± 0.05 ^a c; 2^	0.35 ± 0.61 ^c; 1^	0.00 ± 0.00 ^b; 1^	0.00 ± 0.00 ^b; 2^	0.74 ± 0.02 ^a^
SFA	486.76 ± 10.39 ^a; 1^	633.10 ± 2.26 ^b; 2^	437.95 ± 18.65 ^a; 1^	664.63 ± 40.61 ^b c; 1^	706.10 ± 6.14 ^c; 2^	772.02 ± 6.14 ^d; 1^	353.47 ± 20.27 ^e^
MUFA	1288.49 ± 22.74 ^a; 1^	1686.58 ± 5.06 ^b; 2^	1083.54 ± 29.43 ^c; 1^	1764.35 ± 112.94 ^b c; 1^	1939.23 ± 100.40 ^c; 2^	2151.60 ± 10.86 ^d; 1^	935.21 ± 56.70 ^c^
PUFA	783.41 ± 12.53 ^a c; 1^	830.33 ± 3.15 ^a b; 1^	643.81 ± 25.23 ^c; 1^	945.50 ± 68.10 ^b; 1^	903.54 ± 15.71 ^a b; 1^	994.99 ± 16.56 ^b; 1^	1463.49 ± 110.56 ^d^
n3	53.89 ± 0.72 ^a b; 1^	53.91 ± 0.33 ^b c; 1 2^	41.82 ± 1.28 ^c; 2^	68.55 ± 5.44 ^a; 1^	63.96 ± 11.43 ^a b; 1 2^	69.28 ± 2.26 ^a; 2^	18.77 ± 1.55 ^c^
n6	729.01 ± 11.60 ^a b; 1^	780.49 ± 4.00 ^b; 1^	607.10 ± 24.52 ^a; 1^	890.47 ± 64.02 ^c; 1^	851.87 ± 13.41 ^b c; 1^	939.22 ± 15.27 ^c; 1^	1440.06 ± 109.77 ^d^
Total	2578.82 ± 40.21 ^a; 1^	3166.01 ± 6.10 ^b d; 2^	2178.17 ± 69.17 ^c; 1^	3392.79 ± 211.89 ^d e; 1^	3563.94 ± 132.18 ^e; 2^	3934.89 ± 0.64 ^f; 1^	2760.52 ± 145.25 ^a^

Q: sourdough quinoa; A: sourdough amaranth; BR: sourdough brown rice; QM: sourdough quinoa and *Moringa oleifera*; AM: sourdough amaranth and *Moringa oleifera*; BRM: sourdough amaranth and *Moringa oleifera*; COM: commercial. Different subscript letters indicate significant differences (*p* < 0.005) between columns ^a–f^. SFA: saturated fatty acids; MUFA: monounsaturated fatty acids; PUFA: polyunsaturated fatty acids. Different subscripts indicate differences between samples depending on the sourdough ^1–3^.

**Table 10:** Folate vitamers (FA, THF, 5M-THF, 5F-THF expressed as µg/100 g FW) and total folate (expressed as µg folic acid equivalents/100 g FW).

Sample	Folate Vitaminer				
	FA C_19_H_19_N_7_O_6_	THF C_19_H_23_N_7_O_6_	5M-THF C_20_H_25_N_7_O_6_	5F-THF C_20_H_23_N_7_O_7_	Total Folate Content
QM	122.04 ± 4.52 ^d; 1^	123.31 ± 7.26 ^a; 2^	766.09 ± 12.73 ^b; 1^	384.42 ± 0.99 ^a; 1^	1395.86 ± 14.48 ^a; 1^
AM	882.53 ± 35.98 ^c; 2^	449.17 ± 3.60 ^c; 1^	2660.99 ± 41.51 ^a; 2^	450.89 ± 6.07 ^b; 2^	4443.58 ± 8.00 ^b; 2^
BRM	956.91 ± 11.73 ^c; 3^	280.02 ± 3.71 ^a; 3^	2074.29 ± 29.98 ^c; 2^	493.19 ± 6.87 ^c; 3^	3804.42 ± 36.91 ^c; 3^
Q	35.39 ± 1.04 ^a; 1^	56.95 ± 0.30 ^b; 2^	628.05 ± 20.34 ^d; 1^	274.85 ± 2.94 ^d; 1^	995.25 ± 16.67 ^d; 1^
A	66.79 ± 0.80 ^b; 2^	125.41 ± 4.95 ^a; 1^	2649.11 ± 53.96 ^a; 2^	111.19 ± 1.00 ^e; 2^	2952.50 ± 48.80 ^e; 2^
BR	12.60 ± 0.61 ^a; 3^	104.32 ± 0.21 ^d; 3^	1682.26 ± 27.46 ^e; 2^	345.57 ± 16.97 ^f; 3^	2144.76 ± 9.67 ^f; 3^
COM	25.57 ± 0.68 ^a^	33.86 ± 1.90 ^e^	495.06 ± 4.15 ^f^	85.48 ± 0.59 ^g^	639.96 ± 3.37 ^g^

Q: sourdough quinoa; A: sourdough amaranth; BR: sourdough brown rice; QM: sourdough quinoa and *Moringa oleifera*; AM: sourdough amaranth and *Moringa oleifera*; BRM: amaranth and *Moringa oleifera*; COM: commercial. Different subscript letters indicate significant differences (*p* < 0.005) between samples ^a–g^. FA: folic acid; 5MTHF: 5-methyl-5,6,7,8-tetrahydrofolic acid; THF: 5,6,7,8-tetrahydrofolic acid; 5FTHF: 5-Formyl-5,6,7,8-tetrahydrofolic acid; FW: fresh weight. Different subscripts indicate differences between samples depending on the sourdough ^1–3^.

## Data Availability

The datasets generated for this study are available on request to the corresponding author.
